# A systematic review of spatial habitat associations and modeling of marine fish distribution: A guide to predictors, methods, and knowledge gaps

**DOI:** 10.1371/journal.pone.0251818

**Published:** 2021-05-14

**Authors:** Bradley A. Pickens, Rachel Carroll, Michael J. Schirripa, Francesca Forrestal, Kevin D. Friedland, J. Christopher Taylor

**Affiliations:** 1 CSS-Inc., Fairfax, Virginia, United States of America; 2 NOAA National Centers for Coastal Ocean Science, Beaufort, North Carolina, United States of America; 3 Department of Mathematics and Statistics, University of North Carolina Wilmington, Wilmington, North Carolina, United States of America; 4 Sustainable Fisheries Division, NOAA Fisheries SEFSC, Miami, Florida, United States of America; 5 National Marine Fisheries Service, Narragansett, Rhode Island, United States of America; Technical University of Denmark, DENMARK

## Abstract

As species distribution models, and similar techniques, have emerged in marine ecology, a vast array of predictor variables have been created and diverse methodologies have been applied. Marine fish are vital food resources worldwide, yet identifying the most suitable methodology and predictors to characterize spatial habitat associations, and the subsequent distributions, often remains ambiguous. Our objectives were to identify knowledge gaps in fish guilds, identify research themes, and to determine how data sources, statistics, and predictor variables differ among fish guilds. Data were obtained from an international literature search of peer-reviewed articles (2007–2018; *n =* 225) and research themes were determined based on abstracts. We tested for differences in data sources and modeling techniques using multinomial regressions and used a linear discriminant analysis to distinguish differences in predictors among fish guilds. Our results show predictive studies increased over time, but studies of forage fish, sharks, coral reef fish, and other fish guilds remain sparse. Research themes emphasized habitat suitability and distribution shifts, but also addressed abundance, occurrence, stock assessment, and biomass. Methodologies differed by fish guilds based on data limitations and research theme. The most frequent predictors overall were depth and temperature, but most fish guilds were distinguished by their own set of predictors that focused on their specific life history and ecology. A one-size-fits-all approach is not suitable for predicting marine fish distributions. However, given the paucity of studies for some fish guilds, researchers would benefit from utilizing predictors and methods derived from more commonly studied fish when similar habitat requirements are expected. Overall, the findings provide a guide for determining predictor variables to test and identifies novel opportunities to apply non-spatial knowledge and mechanisms to models.

## Introduction

Species distribution models (SDMs), and similar methods of spatial modeling, have proliferated in the last two decades with the growing availability of geospatial data, increased computational capacity, and new approaches to model building. Spatial modeling approaches have changed dramatically from habitat suitability indices developed in the 1970s [1] to correlative species distribution models [2], shape-constrained models [3], individual-based models [4], mechanistic or physiology-based models [5], bioclimatic envelopes [6], and machine learning techniques with a basis in predictive ability [7]. However, the number of SDMs developed for marine species has lagged behind the effort of terrestrial counterparts, which has been growing rapidly since the 1990s [8]. For terrestrial ecosystems, landscape ecology has been defined as the study of heterogeneous spatial patterns and processes, and it is a product of ecology and geography disciplines [9]. These landscape ecology concepts are now being applied to marine ecosystems in the form of "seascape ecology" [10]. Basic steps of the distribution modeling process, such as assessing the availability and quality of input data, have been outlined in the context of the marine environment [11]. Models of a mixture of marine taxa were evaluated by Melo-Merino et al. [12], but the number of fish studies investigated were limited and the findings emphasized methodology and predictors summarized by treating all taxa as a single unit. Knowledge of marine fish distributions is especially important because of their immense socio-economic value and their use as a food resource. In terms of climate change, the effect of warmer ocean temperatures may not result in immediate changes in presence/absence of fishes, but rather, the physiological constraints of oxygen capacity and demand may initially result in changes in growth, reproduction, 3-dimmensional distribution, and abundance [13]. Therefore, metrics of prediction may differ with fish compared to other marine taxa. Given the unique nature of fish ecology and applications, fish-specific evaluation is needed to identify to determine best practices involving data sources, methodology, and applying the most appropriate predictor variables.

The choice of predictor variables is a central challenge for SDM development [2, 14], and predictors are best derived from sound ecological knowledge and theory of species-environment relationships [15, 16]. The inclusion of too many predictors may result in overfitting, or spurious correlations, while too few predictors may lead to the exclusion of important environmental influences. A vast array of predictor variables have now been developed to quantify the marine physical environment and its biological productivity. Predictor variables may be derived from satellite products, blended-observation satellite products, interpolation of *in situ* or buoy measures, digital elevation models, hindcast ocean models, or earth system models. For example, the online database "Bio-Oracle 2.0" provides worldwide data on 14 oceanographic predictor variables that are described with a minimum, mean, maximum, long-term metrics, and range for three depth zones, totaling 252 variables [17]. Season-specific metrics of ocean characteristics, such as temperature and salinity, may also be applicable. Digital elevation models may be used to produce metrics of depth, slope, aspect, rugosity, slope, slope of slope, curvature, bathymetric position index, and seabed forms at multiple spatial scales [18, 19]. Predictive modeling studies have ranged from those using no predictor variables in favor of kriging geostatistical techniques [20] to those using > 20 different predictor variables [21, 22]. Each of these variables can then be computed at multiple spatial scales [23], which can further increase the number of predictors.

The importance of considering multiple scales of predictors is exemplified by Mannocci et al. [24] who proposed a hierarchy of marine spatial scales, where prey patches determine fine scale habitat associations (1 m –1 km), eddies/fronts/oceanographic features are intermediate in scale (1–100 km), and water masses/currents dictate broad scale (100–1,000 km) associations. Species’ traits, like the mobility of species, will determine responses to spatial and temporal scales of environmental conditions [24]. For instance, Robinson et al. [8] suggests that pelagic species may be well represented by broad-scale environmental data because heterogeneity of their oceanographic environment occurs at a 10–100 km spatial scale, whereas, coastal and benthic species may respond to a finer scale because of local heterogeneity. Traits of marine fish lifespan, age at maturity, fecundity, offspring size, maximum length, growth rate, and trophic level have been used to characterize fish into life-history strategies associated with particular responses to a few variables [25, 26]. For example, Pecuchet et al. [25] characterized short-lived species, such as clupeids, as opportunistic species that have a strong, positive relationship with sea surface temperature (SST), SST seasonality, and chlorophyll-*a*. In contrast, long-lived species responded to depth rather than chlorophyll-a; these species had a moderate, or even a negative association, with SST and its seasonality [25]. Larger species are also more sensitive to temperature change because of physiological constraints imposed by oxygen capacity and demand [27]. Regarding predictor variable differences, Friedland et al. [19] analyzed bottom trawl surveys and found fish functional groups differed in their strength of association with physical, primary productivity, secondary productivity (i.e., zooplankton), and benthic terrain variables. Rather than a one-size-fits-all approach to marine spatial modeling, this information suggests that appropriate predictor variables are likely to differ by fish guild. Here, we define a fish guild as a group of species that utilize similar resources, including requirements for food, shelter, movement, and breeding.

Given the recent growth of marine spatial modeling, the complex array of potential predictor variables available to modelers, the diversity of marine fish species, and evolving statistical methods, we aimed to provide guidance to the development and interpretation of predictive models of marine fish distribution. We conducted a literature synthesis and meta-analysis with the following objectives: 1) identify the most and least frequently studied fish guilds, 2) test how fish data sources, statistical techniques, and predictor variables differ by fish guild, 3) identify common research themes, and 4) discuss implications and recommendations for future modeling of marine fish distributions.

## Materials and methods

### Literature search and scope of the review

We conducted a literature search of peer-reviewed research articles with the Web of Science database. Articles ranging from 2007–2018 were included, and the search was conducted January 16, 2019. The following topic key words were searched within the title, abstract, and key words: "species distribution" OR "ecological niche*" OR "bioclimatic envelope" OR "habitat suitability" OR "habitat model*" OR "spatial distribution" OR "seascape" AND "fish" AND "marine" OR "ocean." We then conducted the same search, but replaced "fish" with "shark" because we found shark articles were better represented with this search. A total of 1,648 articles were obtained from this search. We acknowledge that we were not likely to find 100% of articles related to marine fish predictive modeling, but the search is likely to be representative of such studies.

The review included only fish species, therefore, taxonomic groups such as squid, shrimp, crabs, other crustaceans, corals, and bivalves were not investigated. We limited the review to those research articles that resulted in a spatial prediction of fish distribution beyond fish survey locations. Although studies without spatial predictions may greatly improve our understanding of fish ecology and distribution, ultimately, we were interested in predicting and mapping the distribution of fish over relatively broad areas. No limitations were placed on the dependent variables. Predictions of individual species presence, relative abundance, or relative biomass were considered alongside studies that focused on species richness, species diversity, or similar measures. Of the latter studies, 38 of the 40 studies were based on an accumulation of individual species predictive models. Two studies used species richness directly as a dependent variable, but the methodology of these studies did not differ from the other papers reviewed. For example, predictors focused on temperature and productivity. Given the objectives and scope, we removed the following types of studies from the initial search (see S1 Text for flow diagram): 1) studies that did not include spatial predictions of fish distribution, 2) studies focused solely on the distribution of fishing effort, 3) genetic or evolutionary studies that addressed long-term, broad scale connectivity, 4) studies focused solely on salt marsh, mangrove, intertidal, estuary, or freshwater environments (generally, < 8 psu), 5) simulations of larval dispersal unless spawning was inferred, 6) review or discussion articles, 7) center of gravity studies that did not predict the full distribution of species, and 8) conservation planning studies that used previously developed models. Among the initial set of articles, 225 met the criteria for inclusion.

### Review protocol

For each study, we categorized the fish species into one of ten mutually exclusive guilds (Table 1). The guilds were identified based on fish resource use as well as groups that are often studied and managed together. Sharks, anadromous, coral reef, and hardbottom fish are straightforward. Studies of Chondrichthyan diversity (sharks, rays, chimaeras) were placed under the shark guild. Demersal fish were identified as those using bottom habitats (e.g., cod, *Gadus* spp.), but did not fit into the other categories (e.g., coral reef fish, sharks). Large pelagics included highly mobile fish of high economic value such as tuna and billfish, and medium pelagics included mackerel (Scombridae) or similar-sized pelagic fish. Forage fish are commonly studied as a group, and this guild included species such as anchovy (Engraulidae), sardine and menhaden (Clupeidae). Anadromous fish were excluded from the forage fish guild. The invasive category included all studies of non-native species, and these studies were excluded from being in other fish guilds. Generalized studies included research of two or more of the above fish guilds. These generalized studies often examined species richness, species diversity, or all of the species captured in a particular survey.

**Table 1:** Data sources of marine fish predictive modeling articles attained from the Web of Science database (2007–2018) by fish guild.

Fish guild	*n*	FI (%)	FD (%)	FI and FD (%)	Int. database (%)	Museum, research (%)
*Demersal*	*59*	*93*	*8*	*8*	*7*	*3*
Generalized	40	38**	18	3	40	8
Large pelagic	29	34**	52**	0	3*	3
Shark	19	53**	42*	11	11	5
Forage fish	18	61**	22	11	11	0
Coral reef	16	81	6	6	6	13
Hardbottom	15	93	20	20	1	0
Medium pelagic	15	20**	67**	0	13*	0
Anadromous	7	57**	29	14	14	14
Invasive	6	33**	0	0	33	33
**All studies**		**61**	**25**	**7**	**14**	**5**

Data sources reported are fishery independent (FI), fishery dependent (FD), fishery independent and dependent (FI and FD), international databases (Int. database), and museum or previous research data (Museum, research). Categories are not mutually exclusive.

*p < 0.10,

**p < 0.05,

in a multinomial logistic regression with *demersal fish* used as a reference group and fishery independent, fishery dependent, and international databases tested. Large and medium pelagics, as well as coral reef and hardbottom fish, were combined for statistical analysis.

To quantify fish data sources, we determined if a study used fishery independent, fishery dependent, both fishery independent and dependent, international database, and previous research/museum specimens. Categories were not mutually exclusive. Fishery-dependent data included logbooks, landings, catch observations, incidental catch observations, and recreational catch. International databases included "Sea Around Us," geographic range polygons from "SeaLifebase," and databases of raw species data such as the "Ocean Biogeographic Information System." The modeling, or statistical, technique(s) was recorded and the total number of modeling techniques applied for each study was calculated. For the assessment of statistics, techniques were recorded in the categories of generalized additive model, generalized linear model, Maxent presence-only model, habitat suitability index, multivariate statistic, envelope or bioclimatic envelope models, geostatistics, occupancy model, Bayesian statistic, quantile regression, individual-based models, ordinary least squares, and general machine-learning techniques. We summarized the machine-learning techniques of artificial neural networks, classification and regression trees, random forest, boosted regression trees, support vector machines, and multivariate adaptive regression splines, into a single group of "general machine learning" because of their similarity. Although Maxent modeling is a machine-learning technique, it was treated separately because Maxent is distinctly used for presence-only modeling. We grouped the different methods that applied the principal of bioclimatic envelopes (Surface Range Envelope, AquaMaps, Sea Around Us, bioclimatic envelope). The category of multivariate techniques included ecological niche factor analysis, flexible discriminant analysis, and the non-parametric probabilistic ecological niche model. We did not distinguish models with only fixed effects from those with random effects. Tests of spatial autocorrelation were not recorded except for geostatistical techniques, such as kriging, that directly used autocorrelation for modeling purposes.

We recorded each predictor variable tested, or otherwise utilized (e.g., in habitat suitability indices), in models of fish distribution. For our purposes, the calculations of variables as a mean, minimum, or maximum were considered the same variable (e.g., bottom temperature). However, standard deviation (SD), coefficient of variation (CV), and range were distinguished as a distinct set of variables because they describe heterogeneity or gradients. The wide breadth of habitat descriptors did necessitate some consolidation of predictor variables when the meaning of the predictors were similar (see variable list in S1 Table). Temporal factors (e.g., year, month, day-of-year, day/night) or factors primarily affecting detectability (e.g., moon phase, lunar illumination, clouds, precipitation) were not included because the primary interest was in habitat relationships and identifying the best predictors for mapping fish distribution. We further categorized variables into 1) physiology-based, 2) physical oceanographic, 3) geographic, 4) substrate, and 5) biological (see S2 Table for category of each variable). Physiology-based variables included those that have direct influence on fish physiology. These types of variables are described as direct resource gradients by Austin [28] and included variables such as temperature, salinity, dissolved oxygen, and nutrients. In contrast, physical oceanographic variables are hypothesized to have an indirect effect on fish through enhanced productivity via sea level height anomalies, temperature fronts, chlorophyll-*a*, upwelling events, and ocean currents. Although depth may affect physiology of species, the variable was considered physical oceanographic because it is often a proxy for associations with other physical characteristics. Geographic variables focused on location or proximity to surrounding ecosystems, such as latitude/longitude, distance to shore, distance to shelf, and distance to other ecosystems like mangroves or estuaries. Substrate variables characterized components such as sediment grain size, sessile biota, hardbottom, rugosity, and topography. Biological variables included prey, predators, conspecifics, fishing pressure, competitors, and anthropogenic stress.

To identify common research themes, a word cloud was developed from the abstracts of all articles reviewed. Prior to analysis, we removed numbers, punctuation, common stop words (e.g., pronouns, common verbs), and words that had a generic meaning (S2 Text). Plural words were changed to a singular form. The word cloud depicted the most frequent fifty words, and the R packages ’wordcloud’ [29], ’tm’ [30], and ’stringr’ [31] were used.

### Statistical analyses

We reviewed 225 peer-reviewed scientific articles (S3 Text), and all analyses were conducted using the program R [32]. A single study, Gruss et al. [33], was not included in the predictor variable analysis because they studied 51 groups of fish and invertebrates and each one had a different set of predictor variables. We used a multinomial logistic regression with fish guild as a dependent variable to test for differences in 1) data sources (simplified to fishery-independent, fishery-dependent, and international databases based on sample size), 2) statistical modeling technique, and 3) category of predictor variables (i.e., physiology-based, physical oceanographic, geography, substrate, and biological). The multinomial logistic regression uses a reference group to compare the other groups against. In this case, we selected demersal fish as the reference group for all three tests because they were the most commonly studied fish guild and 2-tailed test were conducted. For tests of fish guilds, we consolidated hardbottom and coral reef fish into a single group of "reef fish." Likewise, we consolidated medium and large pelagic fish into a category of "pelagics." The patterns were very similar within these groups and consolidating improved our sample sizes for statistical testing. For multinomial logistic regression, we used the R package ’nnet’ (v. 7.3–14) [34] and used Wald tests to calculate p-values. Because sample size within fish guilds was relatively small, we report significant differences with an α = 0.10. All values reported are ± 1 SE.

To distinguish specific predictor variables associated with fish guilds, we invoked linear discriminant analysis (LDA), which is a machine-learning analysis used for identifying linear combinations of variables that maximize the separation of known data groupings [35, 36]. We performed LDA with the R packages of ’MASS’ [34], ’irr’ [37], and ’scatterplot3d’ [38]. For this analysis, variables in < 1% of studies were discarded. We first fit an LDA model with a set seed that considered the set of predictor variables and examined the percentage of the trace explained by each of the linear discriminants. We included the appropriate number of discriminants to maintain interpretability and still lead to good separation of the groupings by explaining a sum of at least 75% of the trace. Next, we examined the separation of groups from plots of the discriminants to understand where the groups lie based on the linear combinations of discriminants. To distinguish predictor variables likely responsible for the observed separations, we identified variables with the same combination of discriminant coefficients (positive or negative) that were associated with each fish guild. Finally, we examined agreement statistics including percent agreement and Cohen’s Kappa [39, 40] between the estimated and true groupings to quantify how well the model fit the data.

## Results

### Temporal trends and research themes

**Fig 1:**
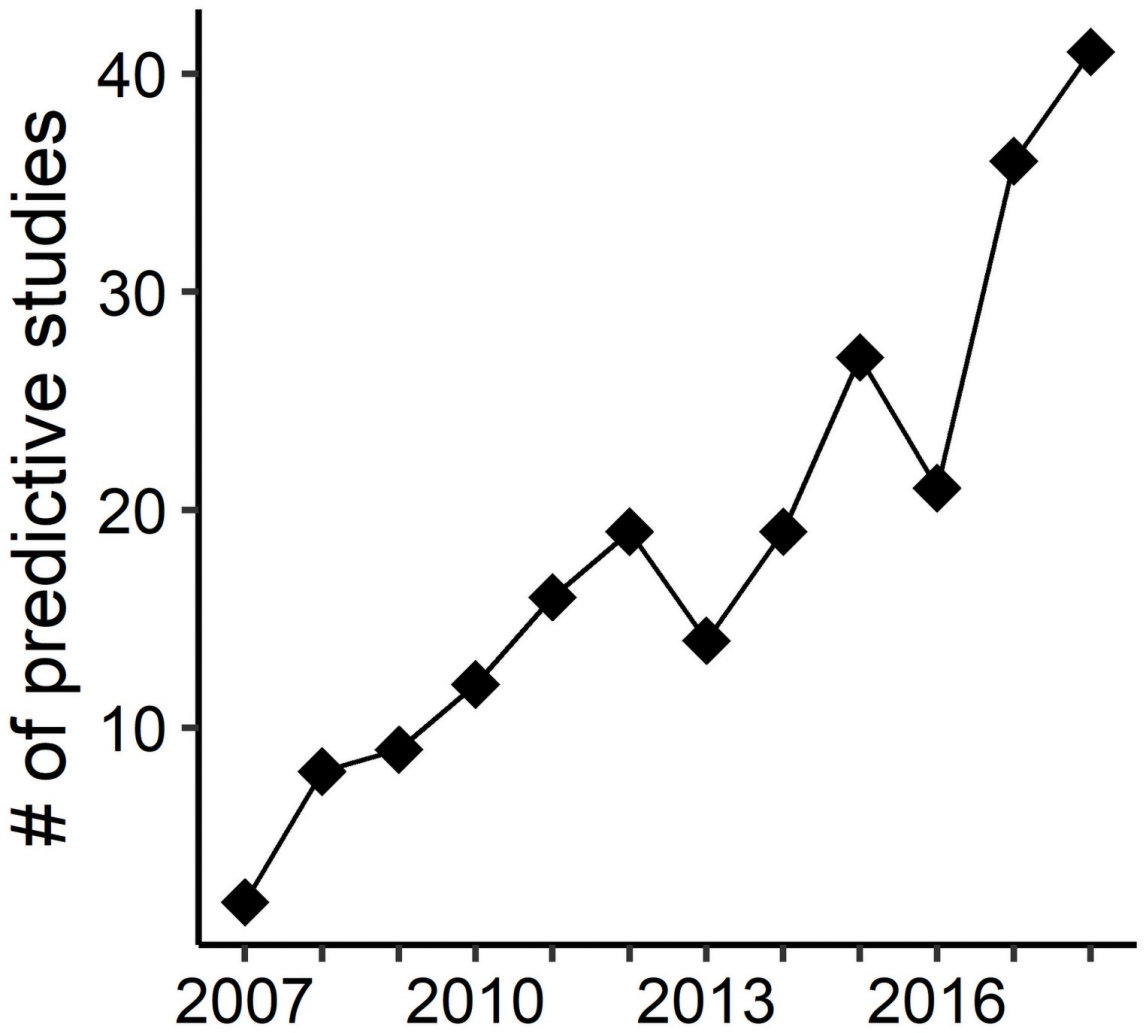
Temporal trend in marine fish predictive modeling studies. Number of published articles from 2007–2018 that predicted the spatial distribution of marine fish based on a search conducted in the Web of Science database.

**Fig 2:**
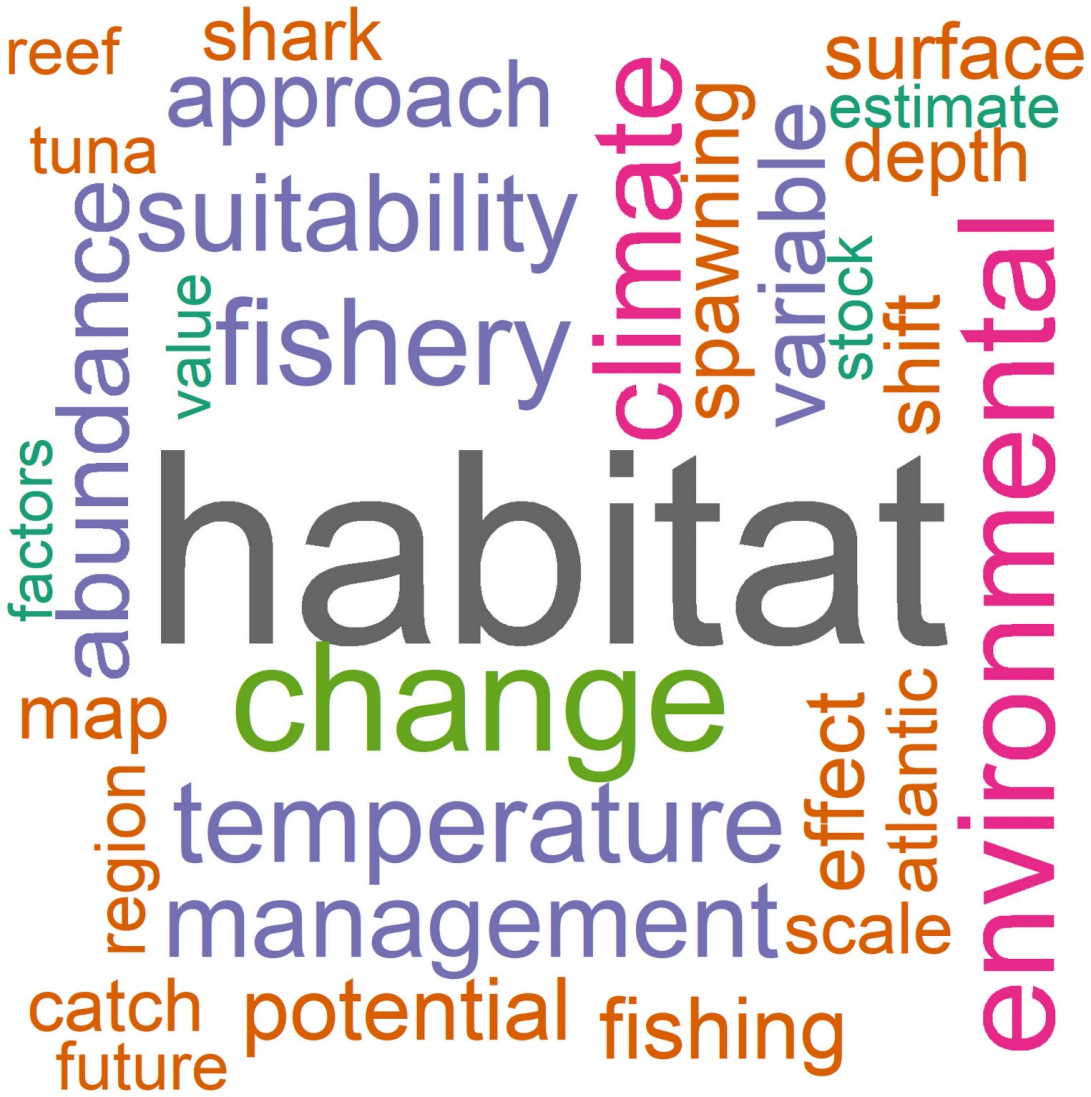
Word cloud representing research themes of marine fish predictive modeling studies. Word size is proportional to its frequency in article abstracts derived from a search of articles from 2007–2018 (*n* = 225).

The number of marine fish predictive modeling articles had a strong upward trend from 2007 to 2018 (Fig 1). Only two such studies were published in 2007, but 41 were published in 2018. Research themes inferred from the word cloud (*n* = 225) (Fig 2) revealed the most frequent terms were *habitat* (frequency = 518), *change* (261), *environmental* (220), *climate* (197), *fishery* (193), *temperature* (176), *suitability* (163), *management* (159), and *abundance* (149) (Fig 2). Other notable terms included *fishing* (116), *conservation* (108), *spawning* (104), *shift* (96), *occurrence* (66), *stock* (64), and *biomass* (63).

### Fish guilds studied, data sources, and modeling methodology

Studies of demersal fish were most common, followed by generalized studies, and large pelagic fish (Table 1). A small number of shark, forage fish, coral reef, hardbottom, and medium pelagic fish studies were conducted; anadromous and invasive studies were rare (Table 1). Notably, the filter-feeding whale shark (*Rhincodon typus*), constituted the subject for 5 of 19 shark studies and tuna (*Thunnus* spp. or *Katsuwonus pelamis*) constituted 20 of 29 large pelagic fish studies. The majority of data sources were fishery-independent surveys (61%) followed by fishery dependent (25%), international databases (14%), fishery independent and dependent surveys (7%), and previous research or museum specimens (5%) (Table 1). Compared to the demersal fish reference group, more research was based on fishery-dependent data for pelagic fish (β = 1.39 ± 0.65) and sharks (β = 1.34 ± 0.74) (Table 1). Studies of invasive species never used fishery-dependent data. Models of invasive species and generalized studies did use international databases extensively, but the differences were not statistically significant. Researchers of pelagic fish used international databases less than demersal studies (β = -2.41 ± 1.08). Studies of demersal fish used more fishery-independent data than all other fish guilds except for reef fish (all p < 0.02).

Of the modeling techniques, generalized additive models (GAMs) were the most frequently used, encompassing 32% of studies (Table 2). Maxent was the most common machine-learning technique with use in 15% of studies followed by general machine-learning analyses. The general machine-learning category included boosted regression trees (6%), classification and regression trees (5%), random forests (4%), multivariate adaptive regression splines (3%), and artificial neural networks (3%). Bayesian statistics, ordinary least squares, individual-based models, quantile regression, and occupancy models were identified, but each accounted for < 5% of studies. Seventeen percent of studies used more than one modeling technique. Compared to the demersal fish reference group, pelagic fish studies used more GAMs (β = 1.06 ± 0.52) and more habitat suitability indices (β = 2.33 ± 0.77) (Table 2). Pelagic fish studies used fewer GLMs (β = -1.29 ± 0.74) and fewer geostatistics (β = -1.94 ± 1.11). Invasive species studies more frequently used habitat suitability indices (β = 2.75 ± 1.47) and Maxent (β = 2.56 ± 1.90), which do not require absences. Reef fish studies less frequently used geostatistics (β = -1.89 ± 1.11). As expected from studies that analyze large numbers of species (e.g., species richness), envelope methods were more frequent in generalized fish studies (β = 3.02 ± 1.03). Some statistical methods were not used with particular fish guilds (Table 2).

**Table 2:** Methodology used to predict the distribution of marine fish in studies spanning 2007–2018 (*n* = 225).

Methodology	% of articles	More frequent than demersal fish	Less frequent than demersal fish
Generalized additive model	32	Pelagics**	NS
Generalized linear model	19	NS	Pelagics*, Invasive (no studies)
Maxent (presence only)	15	Invasive**	Anadromous (no studies)
Habitat suitability index	13	Pelagic***, invasive*	Forage fish (no studies)
General machine learning	11	NS	NS
Multivariate statistics	9	NS	Anadromous (no studies)
Envelope models	8	Generalized***	Reef (no studies)
Geostatistics	7	NS	Pelagics*, reef*, no studies of anadromous, generalized, or invasive

Findings of more frequent and less frequent are from a multinomial logistic regression with demersal fish used as a reference group for comparison. Methods used in > 5% of articles are shown.

*p<0.10,

**p<0.05,

***p<0.01,

****p<0.001.

NS = no significant difference compared to demersal fish studies.

### Predictor variables

**Fig 3:**
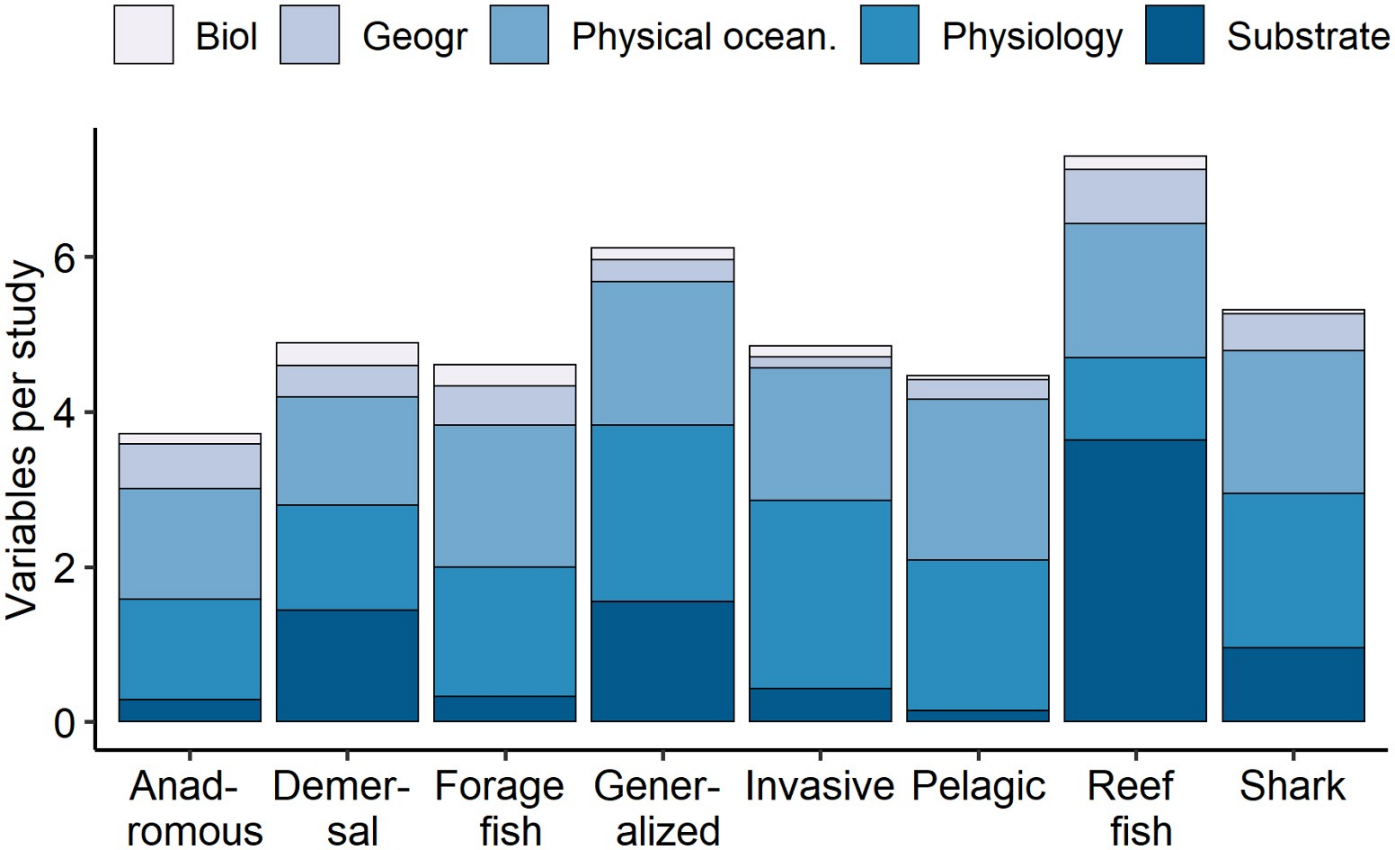
Frequency of predictor variable categories tested by fish guilds. For each fish guild (x-axis), predictor variables were summarized into categories of biological (Biol), geographic (Geogr), physical oceanographic (Physical ocean.), physiology-based oceanographic (Physiology), or substrate (*n* = 224).

We quantified 56 distinct predictor variables that were used in spatial distribution modeling studies of marine fish (*n* = 224 studies) (S2 Table). Of the 1,201 predictors recorded, 32% were physiology-based oceanographic, 32% were physical oceanographic, 25% were substrate, 7% were geographic, and 3% were biological variables. Compared to the demersal fish reference group, biological predictors were less frequent for pelagic fish (β = -1.70 ± 0.77, p = 0.03) (Fig 3). Geography predictors were used more frequently for reef fish (β = 0.69 ± 0.36, p = 0.06). Substrate predictors were less commonly used for forage fish (β = -0.74 ± 0.36, p = 0.04) and pelagic fish (β = -1.34 ± 0.41, p = 0.001). Reef fish were tested with more substrate predictor variables (β = 0.26 ± 0.09, p = 0.003). Physical oceanographic predictors were more common for pelagic fish (β = 0.50 ± 0.19, p = 0.007), and physiology-based predictors were more common for generalized fish studies (β = 0.23 ± 0.12, p = 0.06).

**Fig 4:**
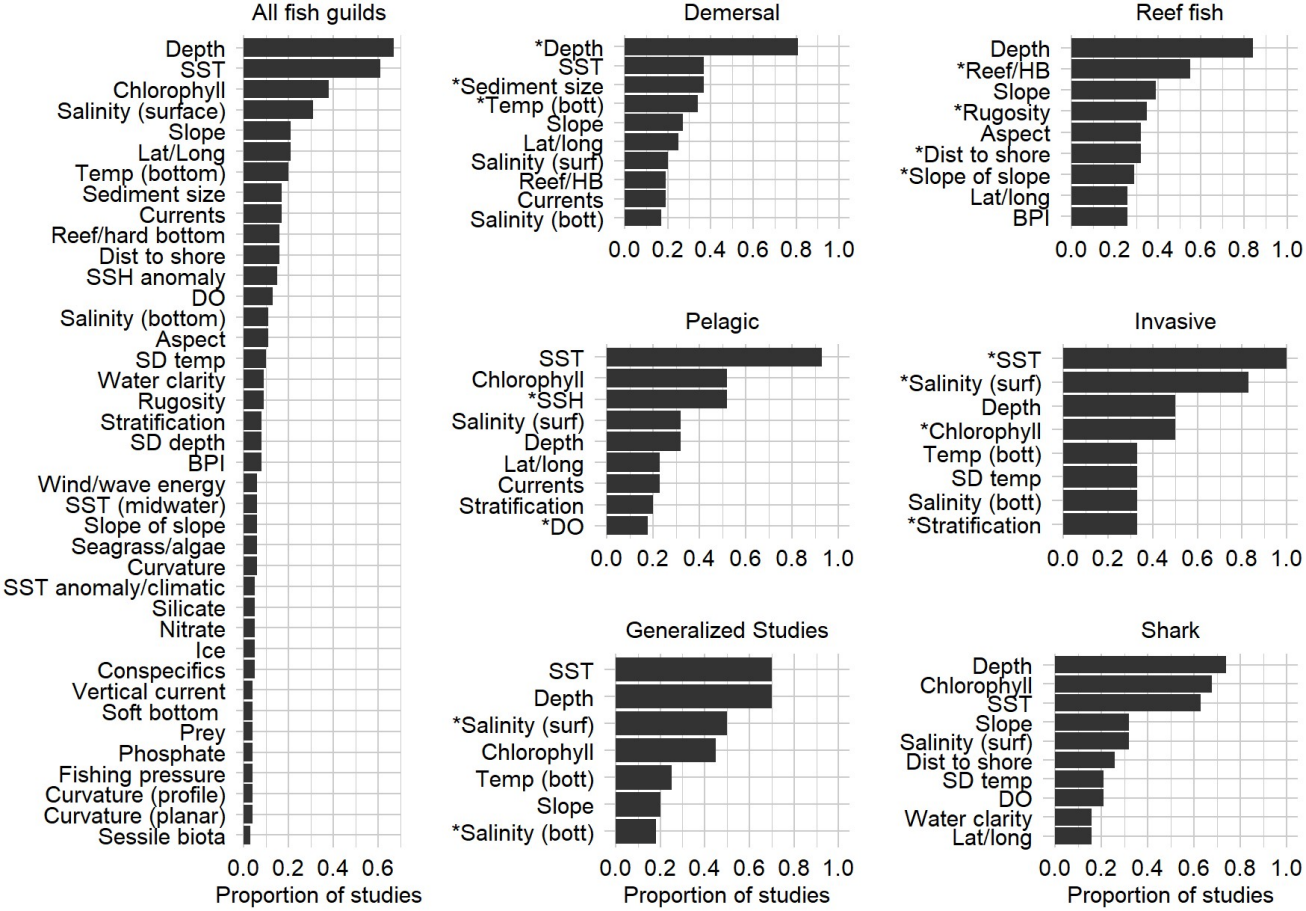
Frequency of predictor variables in marine fish spatial modeling articles. (Left panel) Frequency of predictors among all marine fish studies; variables in ≥ 3% of studies are shown (*n* = 224). (Right panel) The most frequent predictors within each fish guild; only predictors in > 15% of studies, or a maximum of 10 variables, are shown (*n* = 224). * = predictor that distinguished the fish guild from others, as observed in Table 3; BPI = bathymetric position index, dist = distance, DO = dissolved oxygen, HB = hardbottom, Lat/Long = latitude/longitude coordinates, SD = standard deviation, SSH = sea surface height anomaly, SST = sea surface temperature, Temp = temperature, Bott = bottom, Suf = surface.

**Fig 5:**
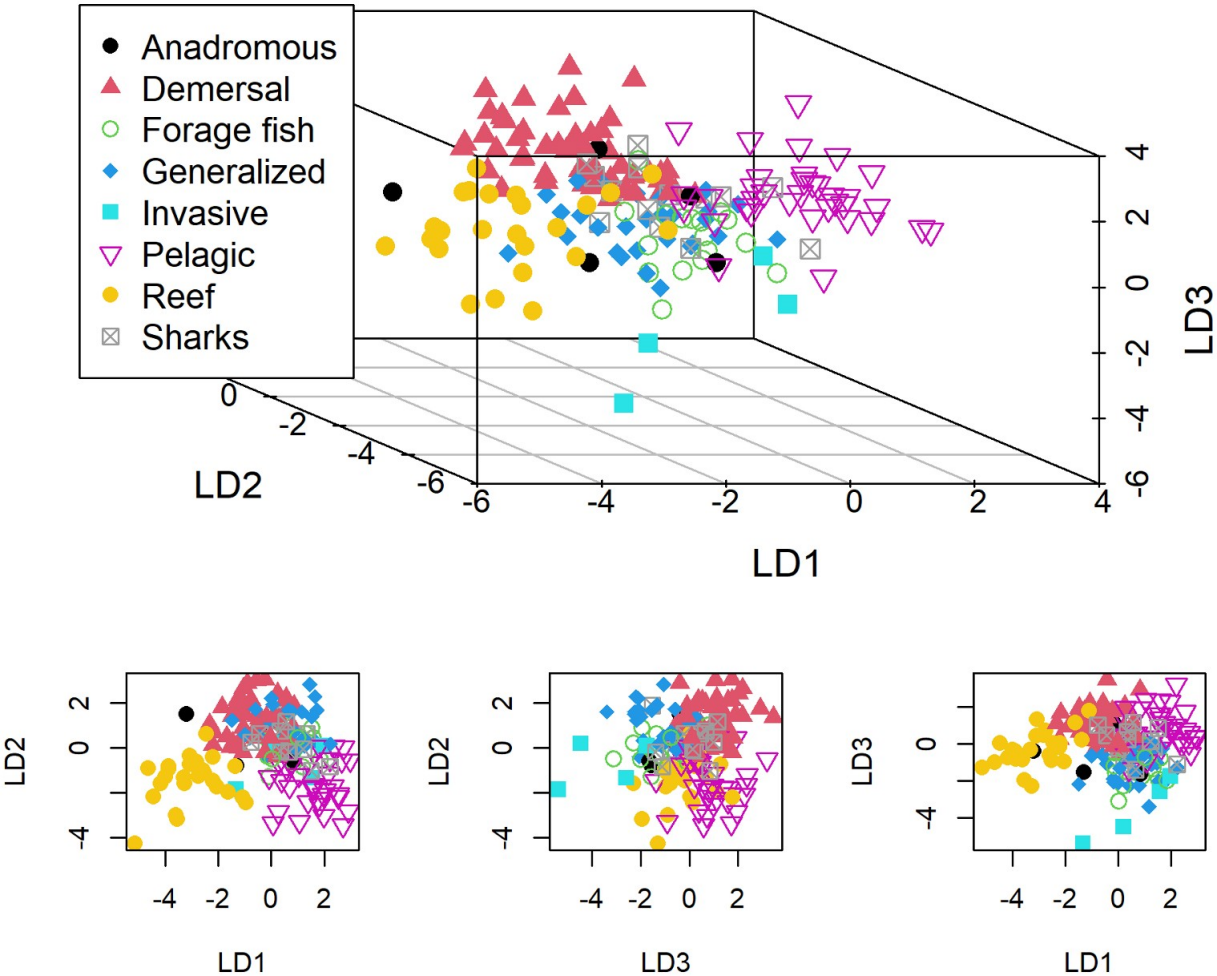
Linear discriminant analysis showing differences in predictor variables tested among fish guilds. The three discriminates (LD1, LD2, LD3) are multivariate combinations of predictor variables tested in 224 marine fish spatial modeling studies. Each fish guild is distinguished by color and shape as depicted in the legend.

Across all studies, depth, SST, chlorophyll, and surface salinity were the most common predictor variables (Fig 4). Furthermore, the frequency distribution of variables by fish guild provides a more detailed assessment of predictor variable applications (Fig 4). The LDA analysis showed that the frequency of predictor variables clearly differed by fish guild (Figs 4 and 5). The three linear discriminants utilized for the separation explained 78% of the trace (39% + 23% + 16%). From these, we observed good separation of demersal, generalized, invasive, pelagic, and reef fish guilds (Fig 5). Anadromous, forage fish, and sharks were not well separated. The overall model fit the data fairly well with percent agreement of 70.1 and Cohen’s Kappa of 0.63 (0.0 being random and 1.0 being perfect agreement). The coefficients from the three discriminants of the LDA model indicate the driving variables for separation of the most separable groups (Table 3). Demersal studies were distinguished by depth, sediment grain size, bottom temperature, particulate organic carbon, distance to (or proportion of) soft bottom, SST anomaly, and habitat type/patch area. Reef fish were distinguished based on anthropogenic stress, water clarity, and substrate attributes of distance to (or proportion of) hardbottom, rugosity, slope of slope, standard deviation of depth, and aspect. Pelagic fish were distinguished by measures of sea surface height anomaly, dissolved oxygen, midwater temperature, phosphate, and pH. Generalized studies were distinguished by consideration of surface and bottom salinity, ice, conspecifics, geology, aspect SD, and curvature profile. Invasive species were represented by variables of SST, chlorophyll, SD of temperature, stratification, and seagrass/macroalgae.

**Table 3:** Distinguishing predictor variables for each marine fish guild based on a linear discriminant analysis.

Variable	LD1	LD2	LD3	Demersal	Reef	Pelagic	Invasive	Generalized
Depth	-0.38	0.63	0.37	X				
SST anomaly	-0.45	0.08	0.79	X				
Bottom temperature	-0.06	0.22	0.61	X				
Particulate organic carbon	-0.51	1.56	0.77	X				
Sediment grain size	-0.26	1.40	1.29	X				
Soft bottom	-0.03	1.78	1.13	X				
Habitat type or patch area	-1.05	0.69	0.78	X				
Slope of slope	-1.72	-2.21	-0.80		X			
Rugosity	-0.69	-0.80	-1.19		X			
SD of depth	-1.04	-0.99	-0.77		X			
Aspect	-0.65	-0.36	-1.14		X			
Reef / hardbottom	-0.74	-1.27	-0.43		X			
Anthropogenic stress	-1.49	-1.18	-0.51		X			
Water clarity	-1.15	-0.42	-2.09		X			
SSH anomaly	0.64	-1.69	1.93			X		
Dissolved oxygen	0.36	-0.31	0.87			X		
SST (midwater)	0.91	-0.06	0.30			X		
Phosphate	5.40	-3.18	1.75			X		
pH	1.66	-2.66	1.58			X		
SST	0.99	-0.54	-0.61				X	
Chlorophyll	0.44	-0.11	-0.60				X	
Stratification	0.88	-0.93	-0.41				X	
Seagrass/macroalgae/algae	0.65	-0.31	-1.15				X	
Temperature SD	0.16	-0.50	-0.26					X
Salinity (surface)	0.30	0.18	-0.59					X
Salinity (bottom)	0.28	0.08	-1.20					X
Curvature (profile)	1.60	2.05	-0.32					X
Aspect SD	0.64	3.43	-1.11					X
Geology	0.43	0.93	-0.38					X
Conspecifics	0.12	0.98	-0.23					X
Ice	0.05	1.38	-1.60					X

Variable loading coefficients are quantified from the three discriminants (LD1, LD2, LD3) (*n* = 224) of a linear discriminant analysis depicting how predictor variables differ by marine fish guilds. "X" indicates the predictor variables that distinguished the most separable fish guilds. Only variables important to the separation of fish guilds are shown.

The median coefficient value of each discriminate (LD1, LD2, and LD3) by fish guild was: anadromous (0.03, 0.51, -0.15), demersal (-0.47, 0.89, 0.70), forage fish (0.66, 0.26, -0.49), generalized (0.58, 0.59, -0.92), invasive (1.27, -0.32, -2.29), pelagic (1.68, -1.68, 0.38), reef fish (-3.09, -1.27, -0.32), and shark (0.35, -0.07, 0.23).

## Discussion

The findings presented here show that methodologies, data sources, and predictors of marine fish are not homogeneous across guilds. Previous research has found that the most frequently expressed goals of marine SDMs are to inform theoretical ecology, current distributions, conservation planning, climate change, and methodology evaluation [11, 12].

However, our investigation found *habitat* was the most frequent theme and was complemented with terms of *suitability* and *environmental*. Our findings concurred with strong interest in *climate*, *temperature*, *change*, and *shift*. The frequency of terms such as *biomass*, *catch*, *population*, *fishery*, *stock*, *management*, *abundance*, and *spawning* suggest a more detailed understanding of distribution may be necessary for fish, and management of fish populations is a key motivator of studies. As examples, Dell et al. [41] estimated how climate change will affect tuna availability and catch, and Hobday et al. [42] aimed to provide seasonal forecasts to fishers. The modeling of fish species abundance and biomass has been conducted for multiple life stages [43–45], and this can aide fishery stock assessments.

Demersal fish were most frequently studied, likely because of their importance as a food resource (e.g., cod, sole, hake, flounder) and the availability of fishery-independent trawl surveys. In comparison, there were relatively few studies of sharks, forage fish, anadromous fish, and reef fish. Melo-Merino et al. [12] found 45% of marine taxa models used the presence-only Maxent technique, but our study found Maxent only accounted for 16% of fish studies. We observed frequent use of GAMs (32%) and machine-learning analyses (Maxent and general = 26%). This likely characterizes the underlying nonlinear responses of fish physiology to environmental conditions such as temperature or dissolved oxygen. Envelope models were more frequently used with generalized studies, and concurrently, 40% of these studies used international databases. This characterizes the common goal of generalized studies to examine broad climate change effects or patterns of species richness, which have less fine-scale management implications. Likewise, invasive species research often used presence-only techniques alongside international databases. The frequent use of habitat suitability indices for pelagic fish likely characterizes knowledge of physiological constraints of species and a lack of absence data.

The selection of predictor variables is a key component for SDM development and interpretation [2]. Predictor variables differed by fish guild in our study and indicates that simply using broadly available data without consideration of fish guild ecology could lead to missing variables of importance. Distinguishing predictors of demersal, reef, and pelagic fish characterized their use of substrate, position in the water column, varying food resource surrogates such as particulate organic carbon and sea surface height anomaly (SSH), physiological restraint of dissolved oxygen for pelagics, and human threats for reef fish. The lack of distinguishing predictors was surprising for forage fish and sharks given the opportunistic life history strategy of forage fish [25] and sharks having distinct traits of large home ranges, low fecundity, high maximum body length, and being at a high trophic level. Both Bradie and Leung [46] and Melo-Merino et al. (2020) found depth, temperature, chlorophyll, salinity, and slope were the most frequent predictors of aquatic and marine taxa. Our findings agree with this assessment, but the most frequent predictors used within fish guilds also included bottom temperature, reef/hardbottom, rugosity, aspect, distance to shore, SSH anomalies, sediment grain size, stratification, currents, and others (Fig 4). Although SDM methodology research has focused primarily on modeling techniques, we concur with Synes and Osborne [47] to suggest that emphasis is needed on the effects of predictor variable choices. They found that models developed from different predictor sets maintained good accuracy, but spatial predictions varied substantially [47]. Our research provides efficient and consistent means to identify the most appropriate predictors of marine fish distributions, while accounting for major differences in fish guild ecology (Fig 4). These results are based on the repeated use of predictor variables by researchers and do not represent an analysis of effect size. As such, these findings can act as a basis for further predictor variable development and may act as a foundation for more detailed meta-analyses on the predictive power of individual variables within fish guilds.

### Implications and recommendations for marine fish modeling

#### 1) Allocate resources to increase use of innovative fishery-independent data sources

Our findings show models of pelagic fish and sharks often rely on fishery-dependent data, yet fishery-independent data provide distinct benefits. For example, demersal fish models benefit from widespread fishery-independent bottom trawl surveys, which have the potential to address climate change and species’ range shifts [48]. The inclusion of zero, or absent, catches through a standardized sampling design offers a substantial advantage over fishery-dependent data. While fishery-dependent data are frequently plentiful and cost-effective, the data may be of coarse resolution or may only be a proxy of fish location. Fishery-dependent data only provide information on where fish were caught or landed, and bias may be introduced because of non-standardized fishing effort or methodology [49, 50]. Fishery-dependent data are presumably from waters with the highest fish densities, and abundance information in suitable habitats where fishing does not occur is lacking. This could affect the derived species-habitat relationships and may be an important knowledge gap for making management decisions. We found the combination of fishery-independent and fishery-dependent data were used in 10–21% of studies of hardbottom fish, sharks, and anadromous fish. In such cases, we recommend the approach of testing for differences in modeling with these two data types [51, 52]. For large pelagics and sharks that cannot be effectively surveyed with fishery-independent surveys, pop-up satellite archival tags (PSAT) have the advantage of recording temperature and tracking movement of fish in four dimensions (latitude, longitude, depth, and across time). For example, Goodyear [53] used PSAT data to create monthly habitat suitability models for Atlantic blue marlin (*Makaira nigricans*) based on temperature that varied by horizontal location and depth; vertical habitat selection differed between diurnal and nocturnal periods. These high resolution data are ideal, but disadvantages include the high cost of tag deployment, risk of failure to collect and/or retrieve the data, and sample size may be relatively small because each tag deployed represents only one fish. Another possible solution is to expand the use of citizen science. Citizen science data has been shown to be as effective as expert surveys for reef fish given some species identification limitations [54], and citizen science data on sharks has recently been used for monitoring [55–57]. Predictive modeling with citizen science data has only begun, but its potential use has been demonstrated by a worldwide analysis of reef fish monitoring data to determine the effect of temperature gradients [58].

#### 2) Consider nonlinear modeling and interactions

Explicitly justify the technique. Extensive testing has been conducted to determine the most accurate SDM techniques [59–61], but the findings are mixed. Our findings suggest that nonlinear species-habitat relationships are expected for marine fish. GAMs and machine-learning analyses provide for flexible, nonlinear response curves, but they can also lead to overfitting, particularly when data gaps exist. To better conform to ecological niche theory, shape-constrained GAMs have been introduced to SDMs [3]. These models constrain response curves to concave functions with the expectation of a unimodal relationship, and the models are particularly applicable when investigating global maxima for variables such as temperature [3]. Given the various spatial scales of predictors influencing marine species’ distributions [24, 62], we suggest explicit consideration of potential interactions or techniques that automatically compute interactions (e.g., boosted regression trees). As an example, selection of fine-scale prey or substrate characteristics may not be possible if broad oceanographic conditions do not allow a species to be present. We recommend explicit justification for selecting a modeling technique, including consideration of objectives, available data, species ecology, number of predictors, and knowledge of potential interactions.

#### 3) Gain perspective from modeling of other fish guilds and use multiscale predictors that are specifically aimed at the ecology of fish guilds

Our study shows clear differences in predictor variables considered among fish guilds, and the findings can act as a guide for variable inclusion. As predictive modeling research is still nascent for several marine fish guilds, there is tremendous potential to learn from studies of other fish guilds. For instance, sharks and large pelagic fish prey on smaller fish, and presumably, select for extremely productive marine environments. However, studies of tuna and other large pelagics are distinguished by a predictor of sea level height anomalies (or fronts), which have rarely been used to predict sharks. Indeed, non-predictive studies of habitat associations have recently found great white shark (*Carcharodon carcharias*) associate with eddies [63], blue shark (*Prionace glauca*) associate with SSH anomalies [64], and three pelagic sharks associate with SST fronts [65]. Incorporating this knowledge with studies of anadromous fish could be beneficial. Anadromous fish research in the marine environment are sparse, but evidence from juvenile Pacific salmon (*Oncorhynchus* spp.) suggests upwelling or downwelling, mixed layer depth, thermal fronts, and prey abundance contribute to salmon distribution, abundance, and stomach fullness [66, 67]. Research has found the decoupling of suitable marine and freshwater habitats can be problematic [68], and anadromous fish research might benefit from methodologies developed for reef fish [69] and marine mammals [70] that account for associations with multiple habitats.

Multiscale predictors, defined as predictors that represent environmental conditions at multiple spatial scales, are recognized as being critical to characterize species’ distributions [24, 71, 72] and warrant consideration. Reef fish research tended to focus on substrate predictors with an emphasis on complexity metrics. However, Pygas et al. [73] found that geographic variables had more predictive power than substrate complexity variables. As examples, distance to shore, distance to shelf edge, and distance to the nearest estuary are all influential predictors of reef fish [18, 74]. In our study, reef fish were the only fish guild without temperature as one of the most frequently tested variables. Waldock et al. [58] found reef fish across the world were more abundant where temperatures are optimal for each species, and Bacheler et al. [75] found hardbottom fish species richness was positively associated with bottom temperature over a span of > 700 km of latitude. Therefore, we suggest further exploration of oceanographic predictors is needed for reef fish, particularly where ocean warming and climate change is forecasted (Asch & Erisman, 2018). In comparison, large pelagic fish studies tend to focus on broad-scale oceanographic predictors with few substrate predictors. These findings may characterize the importance of these features to adults, but requirements of early life stages warrant consideration of fine-scale substrate predictors. For example, juvenile Atlantic bluefin tuna (*Thunnus thynnus*) feed extensively on sand lance (*Ammodytes* spp.) in the mid-Atlantic, USA [76, 77], which are benthic species that are most common in areas with topographic relief [78]. For demersal fish, sediment grain size was a common predictor; this variable often has a relatively minor influence on species’ distribution [19, 21], but such data are often limited and have a coarse resolution.

#### 4) Use fewer proxy variables

SDMs developed with direct resource predictors, characterized as variables having a direct link to species’ physiology [28], are ideal because they enhance the applicability of models to new geographies and can be readily applied to management and conservation, such as identifying species’ habitat requirements. Among all fish, SST and surface salinity were among the four most frequent predictors, yet few fish spend substantial time at the surface. This is particularly poignant for demersal fish, but the 3-D models now developed for large pelagics emphasize the utility of oceanographic measures by depth [53]. These oceanographic models should be further developed and applied to quantify stratification and mixed layer depth, which were common predictors for pelagic fish. Mechanistic biochemical ocean models have been used to develop climate change scenarios for fish by predicting salinity, oxygen, pH, currents, temperature, primary production, and zooplankton over time [79]. These models develop predictors that more directly affect fish rather than only depicting temperature changes. Distance to shore was a frequent proxy variable, and it was particularly common with reef fish and sharks. Although the ecological mechanism for the effect is often not articulated, Olds et al. [69] found 50% of reef fish species were affected by connectivity to mangrove or seagrass, presumably because of adult foraging and juvenile habitat use. Similar studies have concluded salt marsh, mangroves, and seagrass associate with the distribution of reef fish [80, 81]. Coastal bays and estuaries are important nurseries for sharks [82], and Sievers et al. [83] showed that seagrass, and to a lesser extent mangroves, have been associated with a variety of shark life stages. Our review found only one shark study that considered such a variable, distance to mangrove. Given the high productivity of coastal environments, research is needed to evaluate measures of coastal habitats on shark (and other species) distributions.

#### 5) Develop more biological predictors

Biological predictors were considered in only 3% of studies, and prey, conspecifics (e.g., density dependence), and fishing pressure were most common. Prey species are expected to be valuable predictors when there is a mismatch between environmental conditions and prey [8], which might occur because of fishing pressure, temporal dynamics of prey, or when prey correlate with a missing environmental predictor. Recent advances include the Spatial Ecosystem and Population Dynamics Model (SEAPODYM), which is designed to predict pelagic predators based on the predicted distribution of lower and mid-trophic level prey [84, 85]. When tested, copepod abundance has been a useful predictor of mackerel [86] and demersal species [19]. Copepod community shifts due to El Niño events have led to a less lipid-rich community than during other years, and therefore, affects the pelagic food chain [87]. The effects of forage fish abundance on their predators have been mixed [88–90], and new research is needed to examine spatially explicit predator relationships with forage fish. Research on reef fish and demersal fish has shown fishing pressure is important for these groups [91–93], although environmental factors need to be accounted for in models. Researchers should thoughtfully interpret biotic interactions [94]. Nonetheless, Wisz et al. [95] synthesizes solutions to integrate biotic predictors in SDMs, including testing biotic predictors based on supported *a priori* hypotheses, using process-based models, and joint species distribution models that which can account for environmental influences before quantifying associations with biotic variables [96]. Understanding the influence of biotic predictors will improve our understanding of anthropogenic pressures and climate change effects on all fish guilds.

## Conclusions

Knowledge of the current and future distribution of marine fish is critical to food security, economics, and conservation of the world’s fishes. Marine fish SDMs have emerged to map distributions, better understand environmental factors driving habitat suitability, inform management decisions, and to inform policy. We have illuminated knowledge gaps in fish guilds studied as well as identified that predictor variables of fish are not one-size-fits-all. As SDMs of marine fish develop further, there will be new opportunities to integrate new multiscale predictors, quantify complex ecological interactions, and to test existing knowledge on taxonomic groups that are less studied.

## Supporting information

S1 Text The PRISMA flow diagram. (DOC)

S2 Text Commonplace words removed from article abstracts prior to word cloud analysis. (DOCX)

S3 Text List of scholarly papers reviewed on marine fish predictive modeling. (DOCX)

S4 Text PRISMA checklist. (DOC)

S1 Table Consolidated variable names associated with multiple predictors. (DOCX)

S2 Table Predictor variable frequencies and their categorization. Categories were defined for statistical analyses of studies predicting the spatial distribution of marine fish (*n* = 224).
(DOCX)

S1 Appendix Data. (CSV)
