# Supplementary material for: A systematic review of spatial habitat associations and modeling of marine fish distribution: A guide to predictors, methods, and knowledge gaps
Source: PLoS One. 2021 May 14;16(5):e0251818. doi: 10.1371/journal.pone.0251818 (PMC8121303; doi:10.1371/journal.pone.0251818)
Supplement: S1 Text — (DOC) [file pone.0251818.s001.doc]

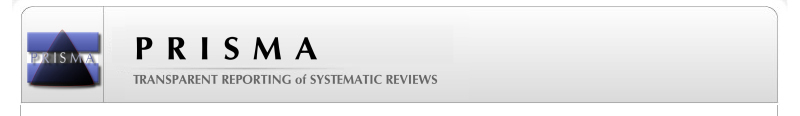
**PRISMA 2009 Flow Diagram**

**S1 Text. The PRISMA flow diagram.**

**Screening**

**Included**

**Eligibility**

**Identification**

Records identified through database searching
(n = 1,648)

Additional records identified through other sources
(n = 0 )

Records after duplicates removed
(n = 1,648)

Records screened
(n = 1,648)

Records excluded
(n = 1,303) Based on criteria stated in manuscript; investigated title/abstract

Full-text articles assessed for eligibility
(n = 345)

Full-text articles excluded, with reasons
(n = 120), criteria for exclusion in the main text.

Studies included in qualitative synthesis
(n = 225)

Studies included in quantitative synthesis (meta-analysis)
(n = 225 except for predictor variable analysis, n = 224)
