# Supplementary material for: A systematic review of spatial habitat associations and modeling of marine fish distribution: A guide to predictors, methods, and knowledge gaps
Source: PLoS One. 2021 May 14;16(5):e0251818. doi: 10.1371/journal.pone.0251818 (PMC8121303; doi:10.1371/journal.pone.0251818)
Supplement: S2 Text — (DOCX) [file pone.0251818.s002.docx]

**S2 Text. Commonplace words removed from article abstracts prior to word cloud analysis.**

"the", "for", "with", "species", "distribution", "distributions","model", "fish", "areas", "models", "within", "likely", "also", "found", "used", "use", "area", "marine", "sea", "north", "one", "using", "many", "however", "southern", "may", "based", "can", "including", "two", "studies", "provide", "large", "across", "eastern", "modeling", "important", "including", "three", "study", "ocean", "modelling", "degrees", "data", "information", "results", "high", "different", "predicted", "predictions", "spatial"
