# Supplementary material for: A systematic review of spatial habitat associations and modeling of marine fish distribution: A guide to predictors, methods, and knowledge gaps
Source: PLoS One. 2021 May 14;16(5):e0251818. doi: 10.1371/journal.pone.0251818 (PMC8121303; doi:10.1371/journal.pone.0251818)
Supplement: S3 Text — (DOCX) [file pone.0251818.s003.docx]

**S3 Text. List of scholarly papers reviewed on marine fish predictive modeling.**

1. Abecasis D, Afonso P, Erzini K. Combining multispecies home range and distribution models aids assessment of MPA effectiveness. Marine Ecology Progress Series. 2014; 513:155-69. http://doi.org/10.3354/meps10987.

2. Adams CF, Alade LA, Legault CM, O'Brien L, Palmer MC, Sosebee KA, et al. Relative importance of population size, fishing pressure and temperature on the spatial distribution of nine Northwest Atlantic groundfish stocks. PLoS One. 2018; 13(4):e0196583. http://doi.org/10.1371/journal.pone.0196583.

3. Adams GD, Flores D, Flores OG, Aarestrup K, Svendsen JC. Spatial ecology of blue shark and shortfin mako in southern Peru: local abundance, habitat preferences and implications for conservation. Endangered Species Research. 2016; 31:19-32. http://doi.org/10.3354/esr00744.

4. Agostini VN, Hendrix AN, Hollowed AB, Wilson CD, Pierce SD, Francis RC. Climate-ocean variability and Pacific hake: a geostatistical modeling approach. Journal of Marine Systems. 2008; 71(3-4):237-48. http://doi.org/10.1016/j.jmarsys.2007.01.010.

5. Alabia ID, Molinos JG, Saitoh SI, Hirawake T, Hirata T, Mueter FJ. Distribution shifts of marine taxa in the Pacific Arctic under contemporary climate changes. Diversity and Distributions. 2018; 24(11):1583-97. http://doi.org/10.1111/ddi.12788.

6. Albouy C, Guilhaumon F, Leprieur F, Lasram FB, Somot S, Aznar R, et al. Projected climate change and the changing biogeography of coastal Mediterranean fishes. Journal of Biogeography. 2013; 40(3):534-47. http://doi.org/10.1111/jbi.12013.

7. Albouy C, Leprieur F, Le Loc'h F, Mouquet N, Meynard CN, Douzery EJP, et al. Projected impacts of climate warming on the functional and phylogenetic components of coastal Mediterranean fish biodiversity. Ecography. 2015; 38(7):681-9. http://doi.org/10.1111/ecog.01254.

8. Alvarez-Berastegui D, Hidalgo M, Tugores MP, Reglero P, Aparicio-Gonzalez A, Ciannelli L, et al. Pelagic seascape ecology for operational fisheries oceanography: modelling and predicting spawning distribution of Atlantic bluefin tuna in Western Mediterranean. ICES Journal of Marine Science. 2016; 73(7):1851-62. http://doi.org/10.1093/icesjms/fsw041.

9. Anadon JD, D'Agrosa C, Gondor A, Gerber LR. Quantifying the spatial ecology of wide-ranging marine species in the Gulf of California: implications for marine conservation planning. PLoS One. 2011; 6(12):e28400. http://doi.org/10.1371/journal.pone.0028400.

10. Asaad I, Lundquist CJ, Erdmann MV, Costello MJ. Delineating priority areas for marine biodiversity conservation in the Coral Triangle. Biological Conservation. 2018; 222:198-211. http://doi.org/10.1016/j.biocon.2018.03.037.

11. Asch RG, Erisman B. Spawning aggregations act as a bottleneck influencing climate change impacts on a critically endangered reef fish. Diversity and Distributions. 2018; 24(12):1712-28. http://doi.org/10.1111/ddi.12809.

12. Authier M, Saraux C, Peron C. Variable selection and accurate predictions in habitat modelling: a shrinkage approach. Ecography. 2017; 40(4):549-60. http://doi.org/10.1111/ecog.01633.

13. Bacheler NM, Bailey KM, Ciannelli L, Bartolino V, Chan KS. Density-dependent, landscape, and climate effects on spawning distribution of walleye pollock *Theragra chalcogramma*. Marine Ecology Progress Series. 2009; 391:1-12. http://doi.org/10.3354/meps08259.

14. Bacheler NM, Ballenger JC. Spatial and temporal patterns of black sea bass sizes and catches in the southeastern United States inferred from spatially explicit nonlinear models. Marine and Coastal Fisheries. 2015; 7(1):523-36. http://doi.org/10.1080/19425120.2015.1095826.

15. Bacheler NM, Schobernd ZH, Berrane DJ, Schobernd CM, Mitchell WA, Teer BZ, et al. Spatial distribution of reef fish species along the southeast US Atlantic Coast inferred from underwater video survey data. PLoS One. 2016; 11(9): e0162653. http://doi.org/10.1371/journal.pone.0162653.

16. Bartolino V, Ciannelli L, Bacheler NM, Chan KS. Ontogenetic and sex-specific differences in density-dependent habitat selection of a marine fish population. Ecology. 2011; 92(1):189-200. http://doi.org/10.1890/09-1129.1.

17. Bartolino V, Ciannelli L, Spencer P, Wilderbuer TK, Chan KS. Scale-dependent detection of the effects of harvesting a marine fish population. Marine Ecology Progress Series. 2012; 444:251-61. http://doi.org/10.3354/meps09434.

18. Bartolino V, Tian HD, Bergstrom U, Jounela P, Aro E, Dieterich C, et al. Spatio-temporal dynamics of a fish predator: density-dependent and hydrographic effects on Baltic Sea cod population. PLoS One. 2017; 12(2):e0172004. http://doi.org/10.1371/journal.pone.0172004.

19. Bassett M, Lindholm J, Garza C, Kvitek R, Wilson-Vandenberg D. Lingcod (*Ophiodon elongatus*) habitat associations in California: implications for conservation and management. Environmental Biology of Fishes. 2018; 101(1):203-13. http://doi.org/10.1007/s10641-017-0692-0.

20. Bauer B, Meier HEM, Casini M, Hoff A, Margonski P, Orio A, et al. Reducing eutrophication increases spatial extent of communities supporting commercial fisheries: a model case study. ICES Journal of Marine Science. 2018; 75(4):1306-17. http://doi.org/10.1093/icesjms/fsy003.

21. Beaugrand G, Kirby RR. Climate, plankton and cod. Global Change Biology. 2010; 16(4):1268-80. http://doi.org/10.1111/j.1365-2486.2009.02063.x.

22. Beaugrand G, Lenoir S, Ibanez F, Mante C. A new model to assess the probability of occurrence of a species, based on presence-only data. Marine Ecology Progress Series. 2011; 424:175-90. http://doi.org/10.3354/meps08939.

23. Beger M, Possingham HP. Environmental factors that influence the distribution of coral reef fishes: modeling occurrence data for broad-scale conservation and management. Marine Ecology Progress Series. 2008; 361:1-13. http://doi.org/10.3354/meps07481.

24. Bernal NA, DeAngelis DL, Schofield PJ, Sealey KS. Predicting spatial and temporal distribution of Indo-Pacific lionfish (*Pterois volitans*) in Biscayne Bay through habitat suitability modeling. Biological Invasions. 2015; 17(6):1603-14. http://doi.org/10.1007/s10530-014-0819-6.

25. Bi HS, Ruppel RE, Peterson WT, Casillas E. Spatial distribution of ocean habitat of yearling Chinook (*Oncorhynchus tshawytscha*) and coho (*Oncorhynchus kisutch*) salmon off Washington and Oregon, USA. Fisheries Oceanography. 2008; 17(6):463-76. http://doi.org/10.1111/j.1365-2419.2008.00493.x.

26. Bouchet PJ, Meeuwig JJ. Drifting baited stereo-videography: a novel sampling tool for surveying pelagic wildlife in offshore marine reserves. Ecosphere. 2015; 6(8). http://doi.org/10.1890/es14-00380.1.

27. Bouchet PJ, Meeuwig JJ, Huang Z, Letessier TB, Nichol SL, Caley MJ, et al. Continental-scale hotspots of pelagic fish abundance inferred from commercial catch records. Global Ecology and Biogeography. 2017; 26(10):1098-111. http://doi.org/10.1111/geb.12619.

28. Boudreau SA, Shackell NL, Carson S, den Heyer CE. Connectivity, persistence, and loss of high abundance areas of a recovering marine fish population in the Northwest Atlantic Ocean. Ecology and Evolution. 2017; 7(22):9739-49. http://doi.org/10.1002/ece3.3495.

29. Brodie S, Hobday AJ, Smith JA, Everett JD, Taylor MD, Gray CA, et al. Modelling the oceanic habitats of two pelagic species using recreational fisheries data. Fisheries Oceanography. 2015; 24(5):463-77. http://doi.org/10.1111/fog.12122.

30. Brodie S, Hobday AJ, Smith JA, Spillman CM, Hartog JR, Everett JD, et al. Seasonal forecasting of dolphinfish distribution in eastern Australia to aid recreational fishers and managers. Deep-Sea Research Part II-Topical Studies in Oceanography. 2017; 140:222-9. http://doi.org/10.1016/j.dsr2.2017.03.004.

31. Brodie S, Litherland L, Stewart J, Schilling HT, Pepperell JG, Suthers IM. Citizen science records describe the distribution and migratory behaviour of a piscivorous predator, *Pomatomus saltatrix*. ICES Journal of Marine Science. 2018; 75(5):1573-82. http://doi.org/10.1093/icesjms/fsy057.

32. Calvert J, McGonigle C, Sethi SA, Harris B, Quinn R, Grabowski J. Dynamic occupancy modeling of temperate marine fish in area-based closures. Ecology and Evolution. 2018; 8(20):10192-205. http://doi.org/10.1002/ece3.4493.

33. Cardinale M, Bartolino V, Llope M, Maiorano L, Skold M, Hagberg J. Historical spatial baselines in conservation and management of marine resources. Fish and Fisheries. 2011; 12(3):289-98. http://doi.org/10.1111/j.1467-2979.2010.00393.x.

34. Carson S, Shackell N, Flemming JM. Local overfishing may be avoided by examining parameters of a spatio-temporal model. PLoS One. 2017; 12(9):e0184427. http://doi.org/10.1371/journal.pone.0184427.

35. Chaalali A, Beaugrand G, Raybaud V, Lassalle G, Saint-Beat B, Le Loc'h F, et al. From species distributions to ecosystem structure and function: a methodological perspective. Ecological Modelling. 2016; 334:78-90. http://doi.org/10.1016/j.ecolmodel.2016.04.022.

36. Champion C, Hobday AJ, Tracey SR, Pecl GT. Rapid shifts in distribution and high-latitude persistence of oceanographic habitat revealed using citizen science data from a climate change hotspot. Global Change Biology. 2018; 24(11):5440-53. http://doi.org/10.1111/gcb.14398.

37. Chang YJ, Sun CL, Chen Y, Yeh SZ, Dinardo G. Habitat suitability analysis and identification of potential fishing grounds for swordfish, *Xiphias gladius*, in the South Atlantic Ocean. International Journal of Remote Sensing. 2012; 33(23):7523-41. http://doi.org/10.1080/01431161.2012.685980.

38. Chang YJ, Sun CL, Chen Y, Yeh SZ, DiNardo G, Su NJ. Modelling the impacts of environmental variation on the habitat suitability of swordfish, *Xiphias gladius*, in the equatorial Atlantic Ocean. ICES Journal of Marine Science. 2013; 70(5):1000-12. http://doi.org/10.1093/icesjms/fss190.

39. Chatfield BS, Van Niel KP, Kendrick GA, Harvey ES. Combining environmental gradients to explain and predict the structure of demersal fish distributions. Journal of Biogeography. 2010; 37(4):593-605. http://doi.org/10.1111/j.1365-2699.2009.02246.x.

40. Chen X, Li G, Feng B, Tian S. Habitat suitability index of Chub mackerel (*Scomber japonicus*) from July to September in the East China Sea. Journal of Oceanography. 2009; 65(1):93-102.

41. Cheung WWL, Dunne J, Sarmiento JL, Pauly D. Integrating ecophysiology and plankton dynamics into projected maximum fisheries catch potential under climate change in the Northeast Atlantic. ICES Journal of Marine Science. 2011; 68(6):1008-18. http://doi.org/10.1093/icesjms/fsr012.

42. Cheung WWL, Jones MC, Lam VWY, Miller DD, Ota Y, Teh L, et al. Transform high seas management to build climate resilience in marine seafood supply. Fish and Fisheries. 2017; 18(2):254-63. http://doi.org/10.1111/faf.12177.

43. Chouinard PM, Dutil JD, Proulx S, Borcard D, Crocker J. A spatial approach to matching marine fish diversity and abundance with habitat features. Hydrobiologia. 2014; 734(1):39-55. http://doi.org/10.1007/s10750-014-1861-1.

44. Ciannelli L, Bartolino V, Chan KS. Non-additive and non-stationary properties in the spatial distribution of a large marine fish population. Proceedings of the Royal Society B-Biological Sciences. 2012; 279(1743):3635-42. http://doi.org/10.1098/rspb.2012.0849.

45. Coelho R, Mejuto J, Domingo A, Yokawa K, Liu KM, Cortes E, et al. Distribution patterns and population structure of the blue shark (*Prionace glauca*) in the Atlantic and Indian Oceans. Fish and Fisheries. 2018; 19(1):90-106. http://doi.org/10.1111/faf.12238.

46. Coggins LG, Bacheler NM, Gwinn DC. Occupancy models for monitoring marine fish: a bayesian hierarchical approach to model imperfect detection with a novel gear combination. PLoS One. 2014; 9(9):e108302. http://doi.org/10.1371/journal.pone.0108302.

47. Colloca F, Garofalo G, Bitetto I, Facchini MT, Grati F, Martiradonna A, et al. The seascape of demersal fish nursery areas in the North Mediterranean Sea, a first step towards the implementation of spatial planning for trawl fisheries. PLoS One. 2015; 10(3):e0119590. http://doi.org/10.1371/journal.pone.0119590.

48. Compton TJ, Morrison MA, Leathwick JR, Carbines GD. Ontogenetic habitat associations of a demersal fish species, *Pagrus auratus*, identified using boosted regression trees. Marine Ecology Progress Series. 2012; 462:219-30. http://doi.org/10.3354/meps09790.

49. Cormon X, Loots C, Vaz S, Vermard Y, Marchal P. Spatial interactions between saithe (*Pollachius virens*) and hake (*Merluccius merluccius*) in the North Sea. ICES Journal of Marine Science. 2014; 71(6):1342-55. http://doi.org/10.1093/icesjms/fsu120.

50. Coro G, Vilas LG, Magliozzi C, Ellenbroek A, Scarponi P, Pagano P. Forecasting the ongoing invasion of *Lagocephalus sceleratus* in the Mediterranean Sea. Ecological Modelling. 2018; 371:37-49. http://doi.org/10.1016/j.ecolmodel.2018.01.007.

51. Costa BHE, Assis J, Franco G, Erzini K, Henriques M, Goncalves EJ, et al. Tropicalization of fish assemblages in temperate biogeographic transition zones. Marine Ecology Progress Series. 2014; 504:241-52. http://doi.org/10.3354/meps10749.

52. Costa TLA, Pennino MG, Mendes LF. Identifying ecological barriers in marine environment: the case study of *Dasyatis marianae*. Marine Environmental Research. 2017; 125:1-9. http://doi.org/10.1016/j.marenvres.2016.12.005.

53. Crec'hriou R, Bonhomme P, Criquet G, Cadiou G, Lenfant P, Bernard G, et al. Spatial patterns and GIS habitat modelling of fish in two French Mediterranean coastal areas. Hydrobiologia. 2008; 612:135-53. http://doi.org/10.1007/s10750-008-9483-0.

54. Cuttitta A, Quinci EM, Patti B, Bonomo S, Bonanno A, Musco M, et al. Different key roles of mesoscale oceanographic structures and ocean bathymetry in shaping larval fish distribution pattern: a case study in Sicilian waters in summer 2009. Journal of Sea Research. 2016; 115:6-17. http://doi.org/10.1016/j.seares.2016.04.005.

55. Dambach J, Rodder D. Applications and future challenges in marine species distribution modeling. Aquatic Conservation-Marine and Freshwater Ecosystems. 2011; 21(1):92-100. http://doi.org/10.1002/aqc.1160.

56. Daqamseh ST, Mansor S, Pradhan B, Billa L, Mahmud AR. Potential fish habitat mapping using MODIS-derived sea surface salinity, temperature and chlorophyll-a data: South China Sea Coastal areas, Malaysia. Geocarto International. 2013; 28(6):546-60.

57. Del Raye G, Weng KC. An aerobic scope-based habitat suitability index for predicting the effects of multi-dimensional climate change stressors on marine teleosts. Deep-Sea Research Part II-Topical Studies in Oceanography. 2015; 113:280-90. http://doi.org/10.1016/j.dsr2.2015.01.014.

58. Delevaux JMS, Jupiter SD, Stamoulis KA, Bremer LL, Wenger AS, Dacks R, et al. Scenario planning with linked land-sea models inform where forest conservation actions will promote coral reef resilience. Scientific Reports. 2018; 8(1):1-21. http://doi.org/10.1038/s41598-018-29951-0.

59. Dell JT, Wilcox C, Matear RJ, Chamberlain MA, Hobday AJ. Potential impacts of climate change on the distribution of longline catches of yellowfin tuna (*Thunnus albacares*) in the Tasman sea. Deep-Sea Research Part II-Topical Studies in Oceanography. 2015; 113:235-45. http://doi.org/10.1016/j.dsr2.2014.07.002.

60. Dell'Apa A, Pennino MG, Bangley CW, Bonzek C. A hierarchical bayesian modeling approach for the habitat distribution of smooth dogfish by sex and season in inshore coastal waters of the US Northwest Atlantic. Marine and Coastal Fisheries. 2018; 10(6):590-605. http://doi.org/10.1002/mcf2.10051.

61. DeVaney SC. Species distribution modeling of deep pelagic eels. Integrative and Comparative Biology. 2016; 56(4):524-30. http://doi.org/10.1093/icb/icw032.

62. Doyle MJ, Debenham C, Barbeaux SJ, Buckley TW, Pirtle JL, Spies IB, et al. A full life history synthesis of arrowtooth flounder ecology in the Gulf of Alaska: exposure and sensitivity to potential ecosystem change. Journal of Sea Research. 2018; 142:28-51. http://doi.org/10.1016/j.seares.2018.08.001.

63. Dueri S, Faugeras B, Maury O. Modelling the skipjack tuna dynamics in the Indian Ocean with APECOSM-E: Part 1. Model formulation. Ecological Modelling. 2012; 245:41-54. http://doi.org/10.1016/j.ecolmodel.2012.02.007.

64. Duffy GA, Chown SL. Explicitly integrating a third dimension in marine species distribution modelling. Marine Ecology Progress Series. 2017; 564:1-8.

65. Dutil JD, Proulx S, Chouinard PM, Borcard D, Larocque R. Distribution and environmental relationships of three species of wolffish (*Anarhichas* spp.) in the Gulf of St. Lawrence. Aquatic Conservation-Marine and Freshwater Ecosystems. 2014; 24(3):351-68. http://doi.org/10.1002/aqc.2370.

66. Elliott SAM, Sabatino AD, Heath MR, Turrell WR, Bailey DM. Landscape effects on demersal fish revealed by field observations and predictive seabed modelling. PLoS One. 2017; 12(12):e0189011. http://doi.org/10.1371/journal.pone.0189011.

67. Eveson JP, Hobday AJ, Hartog JR, Spillman CM, Rough KM. Seasonal forecasting of tuna habitat in the Great Australian Bight. Fisheries Research. 2015; 170:39-49. http://doi.org/10.1016/j.fishres.2015.05.008.

68. Farmer NA, Karnauskas M. Spatial distribution and conservation of speckled hind and warsaw grouper in the Atlantic Ocean off the southeastern U. S. PLoS One. 2013; 8(11):e78682. http://doi.org/10.1371/journal.pone.0078682.

69. Farrell ER, Boustany AM, Halpin PN, Hammond DL. Dolphinfish (*Coryphaena hippurus*) distribution in relation to biophysical ocean conditions in the northwest Atlantic. Fisheries Research. 2014; 151:177-90. http://doi.org/10.1016/j.fishres.2013.11.014.

70. Ferrari R, Malcolm H, Neilson J, Lucieer V, Jordan A, Ingleton T, et al. Integrating distribution models and habitat classification maps into marine protected area planning. Estuarine Coastal and Shelf Science. 2018; 212:40-50. http://doi.org/10.1016/j.ecss.2018.06.015.

71. Fonseca VP, Pennino MG, de Nobrega MF, Oliveira JEL, Mendes LD. Identifying fish diversity hot-spots in data-poor situations. Marine Environmental Research. 2017; 129:365-73. http://doi.org/10.1016/j.marenvres.2017.06.017.

72. Freer JJ, Partridge JC, Tarling GA, Collins MA, Genner MJ. Predicting ecological responses in a changing ocean: the effects of future climate uncertainty. Marine Biology. 2018; 165(1). http://doi.org/10.1007/s00227-017-3239-1.

73. Frelat R, Lindegren M, Denker TS, Floeter J, Fock HO, Sguotti C, et al. Community ecology in 3D: tensor decomposition reveals spatio-temporal dynamics of large ecological communities. PLoS One. 2017; 12(11):e0188205. http://doi.org/10.1371/journal.pone.0188205.

74. French KJ, Shackell NL, den Heyer CE. Strong relationship between commercial catch of adult Atlantic halibut (*Hippoglossus hippoglossus*) and availability of suitable habitat for juveniles in the Northwest Atlantic Ocean. Fishery Bulletin. 2018; 116(2):111-25. http://doi.org/10.7755/fb.116.2.1.

75. Galaiduk R, Radford BT, Harvey ES. Utilizing individual fish biomass and relative abundance models to map environmental niche associations of adult and juvenile targeted fishes. Scientific Reports. 2018; 8(1):1-12. http://doi.org/10.1038/s41598-018-27774-7.

76. Galaiduk R, Radford BT, Saunders BJ, Newman SJ, Harvey ES. Characterizing ontogenetic habitat shifts in marine fishes: advancing nascent methods for marine spatial management. Ecological Applications. 2017; 27(6):1776-88. http://doi.org/10.1002/eap.1565.

77. Garcia-Alegre A, Sanchez F, Gomez-Ballesteros M, Hinz H, Serrano A, Parra S. Modelling and mapping the local distribution of representative species on the Le Danois Bank, El Cachucho Marine Protected Area (Cantabrian Sea). Deep-Sea Research Part II-Topical Studies in Oceanography. 2014; 106:151-64. http://doi.org/10.1016/j.dsr2.2013.12.012.

78. Garcia-Rosello E, Guisande C, Manjarres-Hernandez A, Gonzalez-Dacosta J, Heine J, Pelayo-Villamil P, et al. Can we derive macroecological patterns from primary Global Biodiversity Information Facility data? Global Ecology and Biogeography. 2015; 24(3):335-47. http://doi.org/10.1111/geb.12260.

79. Georgakarakos S, Kitsiou D. Mapping abundance distribution of small pelagic species applying hydroacoustics and Co-Kriging techniques. Hydrobiologia. 2008; 612:155-69. http://doi.org/10.1007/s10750-008-9484-z.

80. Giacomo M, Germana G, Samia F, Okbi R, Othman J, Bachra C, et al. Biomass HotSpot distribution model and spatial interaction of two exploited species of horse mackerel in the south-central Mediterranean Sea. Hydrobiologia. 2018; 821(1):135-50.

81. Gonzales-Andres C, Lopes PFM, Cortes J, Sanchez-Lizaso JL, Pennino MG. Abundance and distribution patterns of *Thunnus albacares* in Isla del Coco National Park through predictive habitat suitability models. PLoS One. 2016; 11(12):e0168212. http://doi.org/10.1371/journal.pone.0168212.

82. Gonzalez-Irusta JM, Wright PJ. Spawning grounds of whiting (*Merlangius merlangus*). Fisheries Research. 2017; 195:141-51. http://doi.org/10.1016/j.fishres.2017.07.005.

83. Gouraguine A, Hidalgo M, Moranta J, Bailey DM, Ordines F, Guijarro B, et al. Elasmobranch spatial segregation in the western Mediterranean. Scientia Marina. 2011; 75(4):653-64. http://doi.org/10.3989/scimar.2011.75n4653.

84. Gruss A, Biggs C, Heyman WD, Erisman B. Prioritizing monitoring and conservation efforts for fish spawning aggregations in the US Gulf of Mexico. Scientific Reports. 2018; 8(1):1-10. http://doi.org/10.1038/s41598-018-26898-0.

85. Gruss A, Chagaris DD, Babcock EA, Tarnecki JH. Assisting ecosystem-based fisheries management efforts using a comprehensive survey database, a large environmental database, and generalized additive models. Marine and Coastal Fisheries. 2018; 10(1):40-70. http://doi.org/10.1002/mcf2.10002.

86. Gruss A, Thorson JT, Babcock EA, Tarnecki JH. Producing distribution maps for informing ecosystem-based fisheries management using a comprehensive survey database and spatio-temporal models. ICES Journal of Marine Science. 2018; 75(1):158-77. http://doi.org/10.1093/icesjms/fsx120.

87. Gruss A, Thorson JT, Sagarese SR, Babcock EA, Karnauskas M, Walter JF, et al. Ontogenetic spatial distributions of red grouper (*Epinephelus mono*) and gag grouper (*Mycteroperca microlepis*) in the US Gulf of Mexico. Fisheries Research. 2017; 193:129-42. http://doi.org/10.1016/j.fishres.2017.04.006.

88. Hacohen-Domene A, Martinez-Rincon RO, Galvan-Magana F, Cardenas-Palomo N, de la Parra-Venegas R, Galvan-Pastoriza B, et al. Habitat suitability and environmental factors affecting whale shark (*Rhincodon typus*) aggregations in the Mexican Caribbean. Environmental Biology of Fishes. 2015; 98(8):1953-64. http://doi.org/10.1007/s10641-015-0413-5.

89. Haghi Vayghan A, Fazli H, Ghorbani R, Lee MA, Nasrollahzadeh Saravi H. Temporal habitat suitability modeling of Caspian shad (*Alosa* spp.) in the southern Caspian Sea. Journal of Limnology. 2016; 75(1):210-23. http://doi.org/10.4081/jlimnol.2015.1215.

90. Hattab T, Albouy C, Lasram FB, Somot S, Le Loc'h F, Leprieur F. Towards a better understanding of potential impacts of climate change on marine species distribution: a multiscale modelling approach. Global Ecology and Biogeography. 2014; 23(12):1417-29. http://doi.org/10.1111/geb.12217.

91. Hattab T, Lasram FB, Albouy C, Sammari C, Romdhane MS, Cury P, et al. The use of a predictive habitat model and a fuzzy logic approach for marine management and planning. PLoS One. 2013; 8(10):e76430. http://doi.org/10.1371/journal.pone.0076430.

92. Hattab T, Leprieur F, Lasram FBR, Gravel D, Le Loc'h F, Albouy C. Forecasting fine-scale changes in the food-web structure of coastal marine communities under climate change. Ecography. 2016; 39(12):1227-37. http://doi.org/10.1111/ecog.01937.

93. Haulsee DE, Breece MW, Brown LM, Wetherbee BM, Fox DA, Oliver MJ. Spatial ecology of *Carcharias taurus* in the northwestern Mid-Atlantic coastal ocean. Marine Ecology Progress Series. 2018; 597:191-206. http://doi.org/10.3354/meps12592.

94. Heinanen S, Chudzinska ME, Mortensen JB, Teo TZE, Utne KR, Sivle LD, et al. Integrated modelling of Atlantic mackerel distribution patterns and movements: a template for dynamic impact assessments. Ecological Modelling. 2018; 387:118-33. http://doi.org/10.1016/j.ecolmodel.2018.08.010.

95. Hill NJ, Tobin AJ, Reside AE, Pepperell JG, Bridge TCL. Dynamic habitat suitability modelling reveals rapid poleward distribution shift in a mobile apex predator. Global Change Biology. 2016; 22(3):1086-96. http://doi.org/10.1111/gcb.13129.

96. Hobday AJ, Hartog JR, Spillman CM, Alves O. Seasonal forecasting of tuna habitat for dynamic spatial management. Canadian Journal of Fisheries and Aquatic Sciences. 2011; 68(5):898-911. http://doi.org/10.1139/f2011-031.

97. Hobday AJ, Hartog JR, Timmiss T, Fielding J. Dynamic spatial zoning to manage southern bluefin tuna (*Thunnus maccoyii*) capture in a multi-species longline fishery. Fisheries Oceanography. 2010; 19(3):243-53. http://doi.org/10.1111/j.1365-2419.2010.00540.x.

98. Hoffle H, Solemdal P, Korsbrekke K, Johannessen M, Bakkeplass K, Kjesbu OS. Variability of northeast Arctic cod (*Gadus morhua*) distribution on the main spawning grounds in relation to biophysical factors. ICES Journal of Marine Science. 2014; 71(6):1317-31. http://doi.org/10.1093/icesjms/fsu126.

99. Hollowed AB, Barbeaux SJ, Cokelet ED, Farley E, Kotwicki S, Ressler PH, et al. Effects of climate variations on pelagic ocean habitats and their role in structuring forage fish distributions in the Bering Sea. Deep-Sea Research Part II-Topical Studies in Oceanography. 2012; 65:230-50. http://doi.org/10.1016/j.dsr2.2012.02.008.

100. Hurst TP, Moss JH, Miller JA. Distributional patterns of 0-group Pacific cod (*Gadus macrocephalus*) in the eastern Bering Sea under variable recruitment and thermal conditions. ICES Journal of Marine Science. 2012; 69(2):163-74. http://doi.org/10.1093/icesjms/fss011.

101. Huse G, Ellingsen I. Capelin migrations and climate change - a modelling analysis. Climatic Change. 2008; 87(1-2):177-97. http://doi.org/10.1007/s10584-007-9347-z.

102. Hussy K, Hinrichsen HH, Huwer B. Hydrographic influence on the spawning habitat suitability of western Baltic cod (*Gadus morhua*). ICES Journal of Marine Science. 2012; 69(10):1736-43. http://doi.org/10.1093/icesjms/fss136.

103. Iampietro PJ, Young MA, Kvitek RG. Multivariate prediction of rockfish habitat suitability in Cordell Bank National Marine Sanctuary and Del Monte Shalebeds, California, USA. Marine Geodesy. 2008; 31(4):359-71.

104. Jones MC, Cheung WWL. Multi-model ensemble projections of climate change effects on global marine biodiversity. ICES Journal of Marine Science. 2015; 72(3):741-52. http://doi.org/10.1093/icesjms/fsu172.

105. Jones MC, Dye SR, Fernandes JA, Frolicher TL, Pinnegar JK, Warren R, et al. Predicting the impact of climate change on threatened species in UK waters. PLoS One. 2013; 8(1):e54216. http://doi.org/10.1371/journal.pone.0054216.

106. Jones MC, Dye SR, Pinnegar JK, Warren R, Cheung WWL. Modelling commercial fish distributions: prediction and assessment using different approaches. Ecological Modelling. 2012; 225:133-45. http://doi.org/10.1016/j.ecolmodel.2011.11.003.

107. Jones MC, Dye SR, Pinnegar JK, Warren R, Cheung WWL. Using scenarios to project the changing profitability of fisheries under climate change. Fish and Fisheries. 2015; 16(4):603-22. http://doi.org/10.1111/faf.12081.

108. Kearney KA, Butler M, Glazer R, Kelble CR, Serafy JE, Stabenau E. Quantifying Florida Bay habitat suitability for fishes and invertebrates under climate change scenarios. Environmental Management. 2015; 55(4):836-56. http://doi.org/10.1007/s00267-014-0336-5.

109. Keyl F. Inter-annual variability in distribution and spatial abundance of sprat, Norway pout and small herring in the North Sea. Hydrobiologia. 2017; 795(1):239-56. http://doi.org/10.1007/s10750-017-3132-4.

110. Kleisner KM, Fogarty MJ, McGee S, Hare JA, Moret S, Perretti CT, et al. Marine species distribution shifts on the US Northeast Continental Shelf under continued ocean warming. Progress in Oceanography. 2017; 153:24-36. http://doi.org/10.1016/j.pocean.2017.04.001.

111. Kostecki C, Rochette S, Girardin R, Blanchard M, Desroy N, Le Pape O. Reduction of flatfish habitat as a consequence of the proliferation of an invasive mollusc. Estuarine Coastal and Shelf Science. 2011; 92(1):154-60. http://doi.org/10.1016/j.ecss.2010.12.026.

112. Kotta J, Nurkse K, Puntila R, Ojaveer H. Shipping and natural environmental conditions determine the distribution of the invasive non-indigenous round goby *Neogobius melanostomus* in a regional sea. Estuarine Coastal and Shelf Science. 2016; 169:15-24. http://doi.org/10.1016/j.ecss.2015.11.029.

113. Koubbi P, Moteki M, Duhamel G, Goarant A, Hulley PA, O'Driscoll R, et al. Ecoregionalization of myctophid fish in the Indian sector of the Southern Ocean: results from generalized dissimilarity models. Deep-Sea Research Part II-Topical Studies in Oceanography. 2011; 58(1-2):170-80. http://doi.org/10.1016/j.dsr2.2010.09.007.

114. Lam VWY, Cheung WWL, Swartz W, Sumaila UR. Climate change impacts on fisheries in West Africa: implications for economic, food and nutritional security. African Journal of Marine Science. 2012; 34(1):103-17. http://doi.org/10.2989/1814232x.2012.673294.

115. Laman EA, Rooper CN, Turner K, Rooney S, Cooper DW, Zimmermann M. Using species distribution models to describe essential fish habitat in Alaska. Canadian Journal of Fisheries and Aquatic Sciences. 2018; 75(8):1230-55. http://doi.org/10.1139/cjfas-2017-0181.

116. Lan KW, Shimada T, Lee MA, Su NJ, Chang Y. Using remote-sensing environmental and fishery data to map potential yellowfin tuna habitats in the tropical Pacific Ocean. Remote Sensing. 2017; 9(5). http://doi.org/10.3390/rs9050444.

117. Lasram FB, Guilhaumon F, Mouillot D. Fish diversity patterns in the Mediterranean Sea: deviations from a mid-domain model. Marine Ecology Progress Series. 2009; 376:253-67. http://doi.org/10.3354/meps07786.

118. Lasram FB, Hattab T, Halouani G, Romdhane MS, Le Loc'h F. Modeling of beta diversity in Tunisian waters: predictions using generalized dissimilarity modeling and bioregionalisation using fuzzy clustering. PLoS One. 2015; 10(7):e0131728. http://doi.org/10.1371/journal.pone.0131728.

119. Leathwick J, Moilanen A, Francis M, Elith J, Taylor P, Julian K, et al. Novel methods for the design and evaluation of marine protected areas in offshore waters. Conservation Letters. 2008; 1(2):91-102. http://doi.org/10.1111/j.1755-263X.2008.00012.x.

120. Leidenberger S, De Giovanni R, Kulawik R, Williams AR, Bourlat SJ. Mapping present and future potential distribution patterns for a meso-grazer guild in the Baltic Sea. Journal of Biogeography. 2015; 42(2):241-54. http://doi.org/10.1111/jbi.12395.

121. Lelievre S, Vaz S, Martin CS, Loots C. Delineating recurrent fish spawning habitats in the North Sea. Journal of Sea Research. 2014; 91:1-14. http://doi.org/10.1016/j.seares.2014.03.008.

122. Lenoir S, Beaugrand G, Lecuyer E. Modelled spatial distribution of marine fish and projected modifications in the North Atlantic Ocean. Global Change Biology. 2011; 17(1):115-29. http://doi.org/10.1111/j.1365-2486.2010.02229.x.

123. Li G, Cao J, Zou XR, Chen XJ, Runnebaum J. Modeling habitat suitability index for Chilean jack mackerel (*Trachurus murphyi*) in the South East Pacific. Fisheries Research. 2016; 178:47-60. http://doi.org/10.1016/j.fishres.2015.11.012.

124. Li G, Chen XJ, Lei L, Guan WJ. Distribution of hotspots of chub mackerel based on remote-sensing data in coastal waters of China. International Journal of Remote Sensing. 2014; 35(11-12):4399-421. http://doi.org/10.1080/01431161.2014.916057.

125. Li M, Zhang CL, Xu BD, Xue Y, Ren YP. Evaluating the approaches of habitat suitability modelling for whitespotted conger (*Conger myriaster*). Fisheries Research. 2017; 195:230-7. http://doi.org/10.1016/j.fishres.2017.07.024.

126. Li YW, Song LM, Nishida T, Gao PF. Development of integrated habitat indices for bigeye tuna, *Thunnus obesus*, in waters near Palau. Marine and Freshwater Research. 2012; 63(12):1244-54. http://doi.org/10.1071/mf12072.

127. Li ZG, Ye ZJ, Wan R, Tanaka KR, Boenish R, Chen Y. Density-independent and density-dependent factors affecting spatio-temporal dynamics of Atlantic cod (*Gadus morhua*) distribution in the Gulf of Maine. ICES Journal of Marine Science. 2018; 75(4):1329-40. http://doi.org/10.1093/icesjms/fsx246.

128. Lin HY, Bush A, Linke S, Possingham HP, Brown CJ. Climate change decouples marine and freshwater habitats of a threatened migratory fish. Diversity and Distributions. 2017; 23(7):751-60. http://doi.org/10.1111/ddi.12570.

129. Loots C, Koubbi P, Duhamel G. Habitat modelling of *Electrona antarctica* (Myctophidae, Pisces) in Kerguelen by generalized additive models and geographic information systems. Polar Biology. 2007; 30(8):951-9. http://doi.org/10.1007/s00300-007-0253-7.

130. Lucifora LO, Barbini SA, Di Giacomo EE, Waessle JA, Figueroa DE. Estimating the geographic range of a threatened shark in a data-poor region: *Cetorhinus maximus* in the South Atlantic Ocean. Current Zoology. 2015; 61(5):811-26. http://doi.org/10.1093/czoolo/61.5.811.

131. Lucifora LO, Garcia VB, Menni RC, Worm B. Spatial patterns in the diversity of sharks, rays, and chimaeras (Chondrichthyes) in the Southwest Atlantic. Biodiversity and Conservation. 2012; 21(2):407-19. http://doi.org/10.1007/s10531-011-0189-7.

132. Lynch PD, Nye JA, Hare JA, Stock CA, Alexander MA, Scott JD, et al. Projected ocean warming creates a conservation challenge for river herring populations. ICES Journal of Marine Science. 2015; 72(2):374-87. http://doi.org/10.1093/icesjms/fsu134.

133. Macdonald JI, Logemann K, Krainski ET, Sigurdsson T, Beale CM, Huse G, et al. Can collective memories shape fish distributions? A test, linking space-time occurrence models and population demographics. Ecography. 2018; 41(6):938-57. http://doi.org/10.1111/ecog.03098.

134. Manderson J, Palamara L, Kohut J, Oliver MJ. Ocean observatory data are useful for regional habitat modeling of species with different vertical habitat preferences. Marine Ecology Progress Series. 2011; 438:1-7. http://doi.org/10.3354/meps09308.

135. Mariani P, MacKenzie BR, Iudicone D, Bozec A. Modelling retention and dispersion mechanisms of bluefin tuna eggs and larvae in the northwest Mediterranean Sea. Progress in Oceanography. 2010; 86(1-2):45-58.

136. Marin-Enriquez E, Seoane J, Muhlia-Melo A. Environmental modeling of occurrence of dolphinfish (*Coryphaena* spp.) in the Pacific Ocean off Mexico reveals seasonality in abundance, hot spots and migration patterns. Fisheries Oceanography. 2018; 27(1):28-40. http://doi.org/10.1111/fog.12231.

137. Marras S, Cucco A, Antognarelli F, Azzurro E, Milazzo M, Bariche M, et al. Predicting future thermal habitat suitability of competing native and invasive fish species: from metabolic scope to oceanographic modelling. Conservation Physiology. 2015; 3(1):1-14. http://doi.org/10.1093/conphys/cou059.

138. Martin CS, Vaz S, Koubbi P, Meaden GJ, Engelhard GH, Lauria V, et al. A digital atlas to link ontogenic shifts in fish spatial distribution to the environment of the eastern English Channel. Dab, *Limanda limanda* as a case-study. Cybium. 2010; 34(1):59-71.

139. Martinez-Rincon RO, Ortega-Garcia S, Vaca-Rodriguez JG, Griffiths SP. Development of habitat prediction models to reduce by-catch of sailfish (*Istiophorus platypterus*) within the purse-seine fishery in the eastern Pacific Ocean. Marine and Freshwater Research. 2015; 66(7):644-53. http://doi.org/10.1071/mf14062.

140. McClanahan TR, Maina JM, Graham NAJ, Jones KR. Modeling reef fish biomass, recovery potential, and management priorities in the western Indian Ocean. PLoS One. 2016; 11(5):e0154585. http://doi.org/10.1371/journal.pone.0154585.

141. McManus MC, Hare JA, Richardson DE, Collie JS. Tracking shifts in Atlantic mackerel (*Scomber scombrus*) larval habitat suitability on the Northeast US Continental Shelf. Fisheries Oceanography. 2018; 27(1):49-62. http://doi.org/10.1111/fog.12233.

142. Mellin C, Bradshaw CJA, Meekan MG, Caley MJ. Environmental and spatial predictors of species richness and abundance in coral reef fishes. Global Ecology and Biogeography. 2010; 19(2):212-22. http://doi.org/10.1111/j.1466-8238.2009.00513.x.

143. Meyers EKM, Tuya F, Barker J, Alvarado DJ, Castro-Hernandez JJ, Haroun R, et al. Population structure, distribution and habitat use of the critically endangered Angelshark, *Squatina squatina*, in the Canary Islands. Aquatic Conservation-Marine and Freshwater Ecosystems. 2017; 27(6):1133-44. http://doi.org/10.1002/aqc.2769.

144. Monk J, Ierodiaconou D, Bellgrove A, Harvey E, Laurenson L. Remotely sensed hydroacoustics and observation data for predicting fish habitat suitability. Continental Shelf Research. 2011; 31(2):S17-S27. http://doi.org/10.1016/j.csr.2010.02.012.

145. Monk J, Ierodiaconou D, Harvey E, Rattray A, Versace VL. Are we predicting the actual or apparent distribution of temperate marine fishes? PLoS One. 2012; 7(4):e34558. http://doi.org/10.1371/journal.pone.0034558.

146. Monk J, Ierodiaconou D, Versace VL, Bellgrove A, Harvey E, Rattray A, et al. Habitat suitability for marine fishes using presence-only modelling and multibeam sonar. Marine Ecology Progress Series. 2010; 420:157-74. http://doi.org/10.3354/meps08858.

147. Moore C, Drazen JC, Radford BT, Kelley C, Newman SJ. Improving essential fish habitat designation to support sustainable ecosystem-based fisheries management. Marine Policy. 2016; 69:32-41. http://doi.org/10.1016/j.marpol.2016.03.021.

148. Moore CH, Harvey ES, Van Niel KP. Spatial prediction of demersal fish distributions: enhancing our understanding of species-environment relationships. ICES Journal of Marine Science. 2009; 66(9):2068-75. http://doi.org/10.1093/icesjms/fsp205.

149. Muhling BA, Brill R, Lamkin JT, Roffer MA, Lee SK, Liu YY, et al. Projections of future habitat use by Atlantic bluefin tuna: mechanistic vs. correlative distribution models. ICES Journal of Marine Science. 2017; 74(3):698-716. http://doi.org/10.1093/icesjms/fsw215.

150. Muhling BA, Lamkin JT, Roffer MA. Predicting the occurrence of Atlantic bluefin tuna (*Thunnus thynnus*) larvae in the northern Gulf of Mexico: building a classification model from archival data. Fisheries Oceanography. 2010; 19(6):526-39. http://doi.org/10.1111/j.1365-2419.2010.00562.x.

151. Muko S, Ohshimo S, Kurota H, Yasuda T, Fukuwaka MA. Long-term change in the distribution of Japanese sardine in the Sea of Japan during population fluctuations. Marine Ecology Progress Series. 2018; 593:141-54. http://doi.org/10.3354/meps12491.

152. Munoz M, Reul A, de Sola LG, Lauerburg RAM, Tello O, Gimpel A, et al. A spatial risk approach towards integrated marine spatial planning: a case study on European hake nursery areas in the North Alboran Sea. Marine Environmental Research. 2018; 142:190-207. http://doi.org/10.1016/j.marenvres.2018.10.008.

153. Murase H, Nagashima H, Yonezaki S, Matsukura R, Kitakado T. Application of a generalized additive model (GAM) to reveal relationships between environmental factors and distributions of pelagic fish and krill: a case study in Sendai Bay, Japan. ICES Journal of Marine Science. 2009; 66(6):1417-24. http://doi.org/10.1093/icesjms/fsp105.

154. Nadon MO, Baum JK, Williams ID, Mcpherson JM, Zgliczynski BJ, Richards BL, et al. Recreating missing population baselines for Pacific reef sharks. Conservation Biology. 2012; 26(3):493-503.

155. Niu M, Jin X, Li X, Wang J. Effects of spatio-temporal and environmental factors on distribution and abundance of wintering anchovy *Engraulis japonicus* in central and southern Yellow Sea. Chinese Journal of Oceanology and Limnology. 2014; 32(3):565-75.

156. Nye JA, Link JS, Hare JA, Overholtz WJ. Changing spatial distribution of fish stocks in relation to climate and population size on the Northeast United States continental shelf. Marine Ecology Progress Series. 2009; 393:111-29.

157. Oh BZL, Sequeira AMM, Meekan MG, Ruppert JLW, Meeuwig JJ. Predicting occurrence of juvenile shark habitat to improve conservation planning. Conservation Biology. 2017; 31(3):635-45. http://doi.org/10.1111/cobi.12868.

158. Okunishi T, Yamanaka Y, Ito S. A simulation model for Japanese sardine (*Sardinops melanostictus*) migrations in the western North Pacific. Ecological Modelling. 2009; 220(4):462-79. http://doi.org/10.1016/j.ecolmodel.2008.10.020.

159. Ono K, Shelton AO, Ward EJ, Thorson JT, Feist BE, Hilborn R. Space-time investigation of the effects of fishing on fish populations. Ecological Applications. 2016; 26(2):392-406. http://doi.org/10.1890/14-1874.

160. Orio A, Bergstrom U, Casini M, Erlandsson M, Eschbaum R, Hussy K, et al. Characterizing and predicting the distribution of Baltic Sea flounder (*Platichthys flesus*) during the spawning season. Journal of Sea Research. 2017; 126:46-55. http://doi.org/10.1016/j.seares.2017.07.002.

161. Ospina-Alvarez A, Bernal M, Catalan IA, Roos D, Bigot JL, Palomera I. Modeling fish egg production and spatial distribution from acoustic data: a step forward into the analysis of recruitment. PLoS One. 2013; 8(9):e73687. http://doi.org/10.1371/journal.pone.0073687.

162. Ottimofiore E, Albouy C, Leprieur F, Descombes P, Kulbicki M, Mouillot D, et al. Responses of coral reef fishes to past climate changes are related to life-history traits. Ecology and Evolution. 2017; 7(6):1996-2005. http://doi.org/10.1002/ece3.2800.

163. Otto SA, Simons S, Stoll JS, Lawson P. Making progress on bycatch avoidance in the ocean salmon fishery using a transdisciplinary approach. ICES Journal of Marine Science. 2016; 73(9):2380-94. http://doi.org/10.1093/icesjms/fsw061.

164. Oyinlola MA, Reygondeau G, Wabnitz CCC, Troell M, Cheung WWL. Global estimation of areas with suitable environmental conditions for mariculture species. PLoS One. 2018; 13(1):e0191086. http://doi.org/10.1371/journal.pone.0191086.

165. Palamara L, Manderson J, Kohut J, Oliver MJ, Gray S, Goff J. Improving habitat models by incorporating pelagic measurements from coastal ocean observatories. Marine Ecology Progress Series. 2012; 447:15-30. http://doi.org/10.3354/meps09496.

166. Palialexis A, Georgakarakos S, Karakassis I, Lika K, Valavanis V. Fish distribution predictions from different points of view: comparing associative neural networks, geostatistics and regression models. Hydrobiologia. 2011; 670(1):165-88.

167. Paramo J, Guillot-Illidge L, Benavides S, Rodriguez A, Sanchez-Ramirez C. Poblational and ecological aspects of demersal fishes in north zone of Colombian Caribbean in relationship with habitat: a tool for identify marine protected areas (mpas) to fisheries management. Caldasia. 2009; 31(1):123-44.

168. Parra HE, Pham CK, Menezes GM, Rosa A, Tempera F, Morato T. Predictive modeling of deep-sea fish distribution in the Azores. Deep-Sea Research Part II-Topical Studies in Oceanography. 2017; 145:49-60. http://doi.org/10.1016/j.dsr2.2016.01.004.

169. Parravicini V, Azzurro E, Kulbicki M, Belmaker J. Niche shift can impair the ability to predict invasion risk in the marine realm: an illustration using Mediterranean fish invaders. Ecology Letters. 2015; 18(3):246-53. http://doi.org/10.1111/ele.12401.

170. Pecuchet L, Reygondeau G, Cheung WWL, Licandro P, van Denderen PD, Payne MR, et al. Spatial distribution of life-history traits and their response to environmental gradients across multiple marine taxa. Ecosphere. 2018; 9(10):e02460. http://doi.org/10.1002/ecs2.2460.

171. Pennino MG, Conesa D, Lopez-Quilez A, Munoz F, Fernandez A, Bellido JM. Fishery-dependent and -independent data lead to consistent estimations of essential habitats. ICES Journal of Marine Science. 2016; 73(9):2302-10. http://doi.org/10.1093/icesjms/fsw062.

172. Pennino MG, Munoz F, Conesa D, Lopez-Quilez A, Bellido JM. Modeling sensitive elasmobranch habitats. Journal of Sea Research. 2013; 83:209-18. http://doi.org/10.1016/j.seares.2013.03.005.

173. Phillips AJ, Ciannelli L, Brodeur RD, Pearcy WG, Childers J. Spatio-temporal associations of albacore CPUEs in the Northeastern Pacific with regional SST and climate environmental variables. ICES Journal of Marine Science. 2014; 71(7):1717-27. http://doi.org/10.1093/icesjms/fst238.

174. Phillips ND, Reid N, Thys T, Harrod C, Payne NL, Morgan CA, et al. Applying species distribution modelling to a data poor, pelagic fish complex: the ocean sunfishes. Journal of Biogeography. 2017; 44(10):2176-87. http://doi.org/10.1111/jbi.13033.

175. Pittman SJ, Brown KA. Multi-scale approach for predicting fish species distributions across coral reef seascapes. PLoS One. 2011; 6(5):e20583. http://doi.org/10.1371/journal.pone.0020583.

176. Pittman SJ, Christensen JD, Caldow C, Menza C, Monaco ME. Predictive mapping of fish species richness across shallow-water seascapes in the Caribbean. Ecological Modelling. 2007; 204(1-2):9-21. http://doi.org/10.1016/j.ecolmodel.2006.12.017.

177. Pittman SJ, Costa BM, Battista TA. Using Lidar bathymetry and boosted regression trees to predict the diversity and abundance of fish and corals. Journal of Coastal Research. 2009; 25(6):27-38. http://doi.org/10.2112/si53-004.1.

178. Rambo H, Stelzenmuller V, Greenstreet SPR, Mollmann C. Mapping fish community biodiversity for European marine policy requirements. ICES Journal of Marine Science. 2017; 74(8):2223-38. http://doi.org/10.1093/icesjms/fsx060.

179. Raybaud V, Bacha M, Amara R, Beaugrand G. Forecasting climate-driven changes in the geographical range of the European anchovy (*Engraulis encrasicolus*). ICES Journal of Marine Science. 2017; 74(5):1288-99. http://doi.org/10.1093/icesjms/fsx003.

180. Rees MJ, Knott NA, Neilson J, Linklater M, Osterloh I, Jordan A, et al. Accounting for habitat structural complexity improves the assessment of performance in no-take marine reserves. Biological Conservation. 2018; 224:100-10. http://doi.org/10.1016/j.biocon.2018.04.040.

181. Reglero P, Ciannelli L, Alvarez-Berastegui D, Balbin R, Lopez-Jurado JL, Alemany F. Geographically and environmentally driven spawning distributions of tuna species in the western Mediterranean Sea. Marine Ecology Progress Series. 2012; 463:273-84. http://doi.org/10.3354/meps09800.

182. Reglero P, Tittensor DP, Alvarez-Berastegui D, Aparicio-Gonzalez A, Worm B. Worldwide distributions of tuna larvae: revisiting hypotheses on environmental requirements for spawning habitats. Marine Ecology Progress Series. 2014; 501:207-24. http://doi.org/10.3354/meps10666.

183. Reynolds SD, Norman BM, Beger M, Franklin CE, Dwyer RG. Movement, distribution and marine reserve use by an endangered migratory giant. Diversity and Distributions. 2017; 23(11):1268-79. http://doi.org/10.1111/ddi.12618.

184. Robinson LM, Hobday AJ, Possingham HP, Richardson AJ. Trailing edges projected to move faster than leading edges for large pelagic fish habitats under climate change. Deep-Sea Research Part II-Topical Studies in Oceanography. 2015; 113:225-34. http://doi.org/10.1016/j.dsr2.2014.04.007.

185. Rochette S, Huret M, Rivot E, Le Pape O. Coupling hydrodynamic and individual-based models to simulate long-term larval supply to coastal nursery areas. Fisheries Oceanography. 2012; 21(4):229-42. http://doi.org/10.1111/j.1365-2419.2012.00621.x.

186. Rooker JR, Simms JR, Wells RJD, Holt SA, Holt GJ, Graves JE, et al. Distribution and habitat associations of billfish and swordfish larvae across mesoscale features in the Gulf of Mexico. PLoS One. 2012; 7(4):e34180. http://doi.org/10.1371/journal.pone.0034180.

187. Runnebaum J, Guan LS, Cao J, O'Brien L, Chen Y. Habitat suitability modeling based on a spatiotemporal model: an example for cusk in the Gulf of Maine. Canadian Journal of Fisheries and Aquatic Sciences. 2018; 75(11):1784-97. http://doi.org/10.1139/cjfas-2017-0316.

188. Salarpouri A, Kamrani E, Kaymaram F, Najafabadi RM. Essential fish habitats (EFH) of small pelagic fishes in the north of the Persian Gulf and Oman Sea, Iran. Iranian Journal of Fisheries Sciences. 2018; 17(1):74-94.

189. Sanchez-Carnero N, Rodriguez-Perez D, Counago E, Le Barzik F, Freire J. Species distribution models and local ecological knowledge in marine protected areas: the case of Os Minarzos (Spain). Ocean & Coastal Management. 2016; 124:66-77. http://doi.org/10.1016/j.ocecoaman.2016.02.008.

190. Sanciangco JC, Carpenter KE, Etnoyer PJ, Moretzsohn F. Habitat availability and heterogeneity and the Indo-Pacific warm pool as predictors of marine species richness in the tropical Indo-Pacific. PLoS One. 2013; 8(2):e56245. http://doi.org/10.1371/journal.pone.0056245.

191. Santora JA, Schroeder ID, Field JC, Wells BK, Sydeman WJ. Spatio-temporal dynamics of ocean conditions and forage taxa reveal regional structuring of seabird-prey relationships. Ecological Applications. 2014; 24(7):1730-47. http://doi.org/10.1890/13-1605.1.

192. Saul SE, Walter JF, Die DJ, Naarc DF, Donahue BT. Modeling the spatial distribution of commercially important reef fishes on the West Florida Shelf. Fisheries Research. 2013; 143:12-20. http://doi.org/10.1016/j.fishres.2013.01.002.

193. Scales KL, Hazen EL, Maxwell SM, Dewar H, Kohin S, Jacox MG, et al. Fit to predict? Eco-informatics for predicting the catchability of a pelagic fish in near real time. Ecological Applications. 2017; 27(8):2313-29. http://doi.org/10.1002/eap.1610.

194. Schmiing M, Afonso P, Tempera F, Santos RS. Predictive habitat modelling of reef fishes with contrasting trophic ecologies. Marine Ecology Progress Series. 2013; 474:201-16. http://doi.org/10.3354/meps10099.

195. Schmiing M, Fontes J, Afonso P. Predictive mapping of reproductive fish habitats to aid marine conservation planning. Canadian Journal of Fisheries and Aquatic Sciences. 2017; 74(7):1016-27. http://doi.org/10.1139/cjfas-2015-0538.

196. Sequeira AMM, Mellin C, Floch L, Williams PG, Bradshaw CJA. Inter-ocean asynchrony in whale shark occurrence patterns. Journal of Experimental Marine Biology and Ecology. 2014; 450:21-9. http://doi.org/10.1016/j.jembe.2013.10.019.

197. Sequeira AMM, Mellin C, Fordham DA, Meekan MG, Bradshaw CJA. Predicting current and future global distributions of whale sharks. Global Change Biology. 2014; 20(3):778-89. http://doi.org/10.1111/gcb.12343.

198. Sequeira AMM, Mellin C, Lozano-Montes HM, Vanderklift MA, Babcock RC, Haywood MDE, et al. Transferability of predictive models of coral reef fish species richness. Journal of Applied Ecology. 2016; 53(1):64-72. http://doi.org/10.1111/1365-2664.12578.

199. Shelton AO, Thorson JT, Ward EJ, Feist BE. Spatial semiparametric models improve estimates of species abundance and distribution. Canadian Journal of Fisheries and Aquatic Sciences. 2014; 71(11):1655-66. http://doi.org/10.1139/cjfas-2013-0508.

200. Siregar ESY, Siregar VP, Agus SB. Fishing ground analysis of yellowfin tuna *Thunnus albacares* in West-Sumatera waters based on GAM model. Jurnal Ilmu Dan Teknologi Kelautan Tropis. 2018; 10(2):501-16. http://doi.org/10.29244/jitkt.v10i2.21908.

201. Sleeman JC, Meekan MG, Wilson SG, Polovina JJ, Stevens JD, Boggs GS, et al. To go or not to go with the flow: environmental influences on whale shark movement patterns. Journal of Experimental Marine Biology and Ecology. 2010; 390(2):84-98. http://doi.org/10.1016/j.jembe.2010.05.009.

202. Song LM, Zhou YQ. Developing an integrated habitat index for bigeye tuna (*Thunnus obesus*) in the Indian Ocean based on longline fisheries data. Fisheries Research. 2010; 105(2):63-74. http://doi.org/10.1016/j.fishres.2010.03.004.

203. Stamoulis KA, Delevaux JMS, Williams ID, Poti M, Lecky J, Costa B, et al. Seascape models reveal places to focus coastal fisheries management. Ecological Applications. 2018; 28(4):910-25. http://doi.org/10.1002/eap.1696.

204. Su NJ, Sun CL, Punt AE, Yeh SZ, DiNardo G. Modelling the impacts of environmental variation on the distribution of blue marlin, *Makaira nigricans*, in the Pacific Ocean. ICES Journal of Marine Science. 2011; 68(6):1072-80. http://doi.org/10.1093/icesjms/fsr028.

205. Su NJ, Sun CL, Punt AE, Yeh SZ, DiNardo G, Chang YJ. An ensemble analysis to predict future habitats of striped marlin (*Kajikia audax*) in the North Pacific Ocean. ICES Journal of Marine Science. 2013; 70(5):1013-22. http://doi.org/10.1093/icesjms/fss191.

206. Swain DP, Benoit HP, Hammill MO. Spatial distribution of fishes in a Northwest Atlantic ecosystem in relation to risk of predation by a marine mammal. Journal of Animal Ecology. 2015; 84(5):1286-98. http://doi.org/10.1111/1365-2656.12391.

207. Thorson JT, Ianelli JN, Kotwicki S. The relative influence of temperature and size-structure on fish distribution shifts: a case-study on Walleye pollock in the Bering Sea. Fish and Fisheries. 2017; 18(6):1073-84. http://doi.org/10.1111/faf.12225.

208. Thorson JT, Pinsky ML, Ward EJ. Model-based inference for estimating shifts in species distribution, area occupied and centre of gravity. Methods in Ecology and Evolution. 2016; 7(8):990-1002. http://doi.org/10.1111/2041-210x.12567.

209. Thorson JT, Rindorf A, Gao J, Hanselman DH, Winker H. Density-dependent changes in effective area occupied for sea-bottom-associated marine fishes. Proceedings of the Royal Society B-Biological Sciences. 2016; 283(1840):20161853. http://doi.org/10.1098/rspb.2016.1853.

210. Tolimieri N, Shelton AO, Feist BE, Simon V. Can we increase our confidence about the locations of biodiversity 'hotspots' by using multiple diversity indices? Ecosphere. 2015; 6(12):1-13. http://doi.org/10.1890/es14-00363.1.

211. Townhill B, Pinnegar J, Tinker J, Jones M, Simpson S, Stebbing P, et al. Non-native marine species in north-west Europe: developing an approach to assess future spread using regional downscaled climate projections. Aquatic Conservation-Marine and Freshwater Ecosystems. 2017; 27(5):1035-50. http://doi.org/10.1002/aqc.2764.

212. Tseng CT, Su NJ, Sun CL, Punt AE, Yeh SZ, Liu DC, et al. Spatial and temporal variability of the Pacific saury (*Cololabis saira*) distribution in the northwestern Pacific Ocean. ICES Journal of Marine Science. 2013; 70(5):991-9. http://doi.org/10.1093/icesjms/fss205.

213. Turner SM, Hare JA, Manderson JP, Richardson DE, Hoey JJ. Evaluation of species distribution forecasts: a potential predictive tool for reducing incidental catch in pelagic fisheries. Canadian Journal of Fisheries and Aquatic Sciences. 2017; 74(11):1717-31. http://doi.org/10.1139/cjfas-2016-0274.

214. Vaz S, Martin CS, Eastwood PD, Ernande B, Carpentier A, Meaden GJ, et al. Modelling species distributions using regression quantiles. Journal of Applied Ecology. 2008; 45(1):204-17. http://doi.org/10.1111/j.1365-2664.2007.01392.x.

215. Veiga N, Moura T, Figueiredo I. Spatial overlap between the leafscale gulper shark and the black scabbardfish off Portugal. Aquatic Living Resources. 2013; 26(4):343-53. http://doi.org/10.1051/alr/2013070.

216. Wabnitz CCC, Lam VWY, Reygondeau G, Teh LCL, Al-Abdulrazzak D, Khalfallah M, et al. Climate change impacts on marine biodiversity, fisheries and society in the Arabian Gulf. PLoS One. 2018; 13(5):e0194537. http://doi.org/10.1371/journal.pone.0194537.

217. Wang LF, Kerr LA, Record NR, Bridger E, Tupper B, Mills KE, et al. Modeling marine pelagic fish species spatiotemporal distributions utilizing a maximum entropy approach. Fisheries Oceanography. 2018; 27(6):571-86. http://doi.org/10.1111/fog.12279.

218. Ward EJ, Jannot JE, Lee YW, Ono K, Shelton AO, Thorson JT. Using spatiotemporal species distribution models to identify temporally evolving hotspots of species co-occurrence. Ecological Applications. 2015; 25(8):2198-209. http://doi.org/10.1890/15-0051.1.sm.

219. Weatherdon LV, Ota Y, Jones MC, Close DA, Cheung WWL. Projected scenarios for coastal First Nations' fisheries catch potential under climate change: management challenges and opportunities. PLoS One. 2016; 11(1):e0145285. http://doi.org/10.1371/journal.pone.0145285.

220. Yen KW, Lu HJ, Chang Y, Lee MA. Using remote-sensing data to detect habitat suitability for yellowfin tuna in the Western and Central Pacific Ocean. International Journal of Remote Sensing. 2012; 33(23):7507-22. http://doi.org/10.1080/01431161.2012.685973.

221. Yen KW, Wang GH, Lu HJ. Evaluating habitat suitability and relative abundance of skipjack (*Katsuwonus pelamis*) in the Western and Central Pacific during various El Nino events. Ocean & Coastal Management. 2017; 139:153-60. http://doi.org/10.1016/j.ocecoaman.2017.02.011.

222. Young M, Carr MH. Application of species distribution models to explain and predict the distribution, abundance and assemblage structure of nearshore temperate reef fishes. Diversity and Distributions. 2015; 21(12):1428-40. http://doi.org/10.1111/ddi.12378.

223. Young MA, Iampietro PJ, Kvitek RG, Garza CD. Multivariate bathymetry-derived generalized linear model accurately predicts rockfish distribution on Cordell Bank, California, USA. Marine Ecology Progress Series. 2010; 415:247-61. http://doi.org/10.3354/meps08760.

224. Yu W, Guo A, Zhang Y, Chen XJ, Qian WG, Li YS. Climate-induced habitat suitability variations of chub mackerel *Scomber japonicus* in the East China Sea. Fisheries Research. 2018; 207:63-73. http://doi.org/10.1016/j.fishres.2018.06.007.

225. Zhang X, Vincent ACJ. Integrating multiple datasets with species distribution models to inform conservation of the poorly-recorded Chinese seahorses. Biological Conservation. 2017; 211:161-71. http://doi.org/10.1016/j.biocon.2017.05.020.
