# Supplementary material for: A systematic review of spatial habitat associations and modeling of marine fish distribution: A guide to predictors, methods, and knowledge gaps
Source: PLoS One. 2021 May 14;16(5):e0251818. doi: 10.1371/journal.pone.0251818 (PMC8121303; doi:10.1371/journal.pone.0251818)
Supplement: S1 Table — (DOCX) [file pone.0251818.s005.docx]

**S1 Table. Consolidated variable names associated with multiple predictors.**

**Consolidated variable name Predictor variables included**

Anthropogenic stress Vessel traffic, distance to market or port, market population, ocean acidification

Bed sheer stress Wave orbital velocity

Bathymetric position index Hypsometric index, relief, topographic index

Chlorophyll-*a* Methods of estimating primary productivity and phytoplankton

Conspecifics Density dependence, including positive (e.g., spawning) or negative associations over time

Current velocity/direction U- and V-direction current velocity, current direction, divergence, convergence, current anomaly, distance to current

Curvature planar SD of curvature planar

Curvature profile SD of curvature profile

Depth SD Coefficient of variation of depth, depth range

Distance to estuary or river Distance to canal, distance to strait

Fishing pressure Fishing effort, management, protection

Geology (general) Features such as canyons, sea mounts

Habitat type or patch area Number of patches, area of specific habitat, categories of specific habitat (e.g., coral atolls)

Hardbottom / reef Distance to hardbottom or reef, proportion of area as hardbottom or reef

Stratification Mixed layer depth, upwelling metrics such as cold water mass

Sea surface height anomaly Kinetic energy, eddy development, SD of sea surface height, thermal fronts as a derivative of sea surface height, vorticity

Soft bottom Proportion of soft bottom or distance to soft bottom

SST anomaly Climatic variables (e.g., El Nino)

Slope of slope SD of slope

Temperature SD Water temperature range or gradient

Water clarity Turbidity, euphotic depth, light attenuation, photosynthetic active radiation

Wind or wave energy Fetch, exposure, wave energy
