# Supplementary material for: A systematic review of spatial habitat associations and modeling of marine fish distribution: A guide to predictors, methods, and knowledge gaps
Source: PLoS One. 2021 May 14;16(5):e0251818. doi: 10.1371/journal.pone.0251818 (PMC8121303; doi:10.1371/journal.pone.0251818)
Supplement: S2 Table — Categories were defined for statistical analyses of studies predicting the spatial distribution of marine fish (n = 224). (DOCX) [file pone.0251818.s006.docx]

**S2 Table. Predictor variable frequencies and their categorization.** Categories were defined for statistical analyses of studies predicting the spatial distribution of marine fish (*n* = 224).

**Predictor variable Proportion of articles Category**

Depth 0.67 Physical oceanographic

Sea surface temperature 0.61 Physiology-based

Chlorophyll-*a* 0.38 Physical oceanographic

Surface salinity 0.31 Physiology-based

Slope 0.21 Substrate

Latitude/longitude 0.21 Geographic

Bottom temperature 0.20 Physiology-based

Sediment grain size 0.17 Substrate

Current velocity/direction 0.17 Physical oceanographic

Hard bottom / reef 0.16 Substrate

Distance to shore 0.16 Geographic

Sea surface height anomaly 0.15 Physical oceanographic

Dissolved oxygen 0.13 Physiology-based

Aspect 0.12 Substrate

Bottom salinity 0.11 Physiology-based

Temperature SD 0.10 Physical oceanographic

Rugosity 0.09 Substrate

Water clarity 0.09 Physiology-based

Depth SD 0.08 Substrate

Bathymetric position index 0.08 Substrate

Stratification 0.08 Physical oceanographic

Slope of slope 0.06 Substrate

Seagrass/macroalgae/algae 0.06 Substrate

Wind or wave energy 0.06 Physical oceanographic

Temperature- midwater 0.06 Physiology-based

Nitrate 0.05 Physiology-based

Ice 0.05 Physical oceanographic

Curvature 0.05 Substrate

Silicate 0.05 Physiology-based

Conspecifics 0.05 Biological

SST anomaly 0.05 Physical oceanographic

Prey 0.04 Biological

Curvature planar 0.04 Substrate

Phosphate 0.04 Physiology-based

Proportion or distance to soft bottom 0.04 Substrate

Curvature profile 0.04 Substrate

Fishing pressure 0.04 Biological

**S3 Appendix. Continued.**

**Variable Proportion of articles Category**

Bed sheer stress 0.04 Physical oceanographic

Sessile biota 0.03 Substrate

Carbonate or calcite 0.02 Physiology-based

Particulate organic carbon 0.02 Physiology-based

Habitat type or patch area 0.02 Substrate

Geology (general) 0.02 Substrate

Fractal dimension 0.02 Substrate

Other substrate measure 0.02 Substrate

pH 0.02 Physiology-based

Competitor 0.01 Biological

Anthropogenic stress 0.01 Biological

Distance to estuary or river 0.01 Geographic

Aspect SD 0.01 Substrate

Predation risk 0.01 Biological

Distance to mangrove 0.01 Geographic

Iron 0.00 Physiology-based

Distance to shelf 0.00 Geographic

Substrate diversity 0.00 Substrate
